# Unexpected diversity and ecological significance of uncultivable large virus-like particles in aquatic environments

**DOI:** 10.1093/ismeco/ycaf098

**Published:** 2025-06-05

**Authors:** Hermine Billard, Maxime Fuster, François Enault, Jean-François Carrias, Léa Fargette, Margot Carrouée, Perrine Desmares, Tom O Delmont, Pauline Nogaret, Estelle Bigeard, Gwenn Tanguy, Anne-Claire Baudoux, Urania Christaki, Télesphore Sime-Ngando, Jonathan Colombet

**Affiliations:** Laboratoire Microorganismes : Génome et Environnement (LMGE), UMR CNRS 6023, Université Clermont-Auvergne, F-63000 Clermont-Ferrand, France; Laboratoire Microorganismes : Génome et Environnement (LMGE), UMR CNRS 6023, Université Clermont-Auvergne, F-63000 Clermont-Ferrand, France; Laboratoire Microorganismes : Génome et Environnement (LMGE), UMR CNRS 6023, Université Clermont-Auvergne, F-63000 Clermont-Ferrand, France; Laboratoire Microorganismes : Génome et Environnement (LMGE), UMR CNRS 6023, Université Clermont-Auvergne, F-63000 Clermont-Ferrand, France; Laboratoire Microorganismes : Génome et Environnement (LMGE), UMR CNRS 6023, Université Clermont-Auvergne, F-63000 Clermont-Ferrand, France; Laboratoire Microorganismes : Génome et Environnement (LMGE), UMR CNRS 6023, Université Clermont-Auvergne, F-63000 Clermont-Ferrand, France; Laboratoire Microorganismes : Génome et Environnement (LMGE), UMR CNRS 6023, Université Clermont-Auvergne, F-63000 Clermont-Ferrand, France; Génomique Métabolique, Genoscope, Institut François Jacob, CEA, CNRS, Univ. Evry, Université Paris-Saclay, Evry, France; Sorbonne Université CNRS, Station Biologique de Roscoff, UMR, 7144, Roscoff, France; Sorbonne Université CNRS, Station Biologique de Roscoff, UMR, 7144, Roscoff, France; Sorbonne Université CNRS, Station Biologique de Roscoff, FR2424, Roscoff, France; Sorbonne Université CNRS, Station Biologique de Roscoff, UMR, 7144, Roscoff, France; UMR CNRS 8187 LOG, Université Littoral Côte d’Opale, Université de Lille, Wimereux, France; Laboratoire Microorganismes : Génome et Environnement (LMGE), UMR CNRS 6023, Université Clermont-Auvergne, F-63000 Clermont-Ferrand, France; Laboratoire Microorganismes : Génome et Environnement (LMGE), UMR CNRS 6023, Université Clermont-Auvergne, F-63000 Clermont-Ferrand, France

**Keywords:** giant virus, jumbo phages, viral ecology, viral diversity

## Abstract

The discovery of jumbo phages and giant viruses of microeukaryotes has transformed our perception of the virosphere. Metagenomic and metatranscriptomic data further highlight their diversity and ecological impact. Nevertheless, sequence-based approaches fail to take into account the morphological diversity of non-cultivated viruses, resulting in our fragmented understanding of their nature and role in the environment. Here, we combined flow cytometry and electron microscopy to uncover both previously unsuspected morphological diversity and significant abundances of large virus-like particles in aquatic environments. We discovered new viral morphotypes, all likely to be associated with microeukaryotes. We also obtained insights into the multi-year dynamics of the abundances of both giant microeukaryotic virus-like particles and jumbo phage-like particles. This work deepens our understanding of large virus and reveals their key role as regulators of microbial communities.

## Graphical Abstract

**Graphical Abstract:**
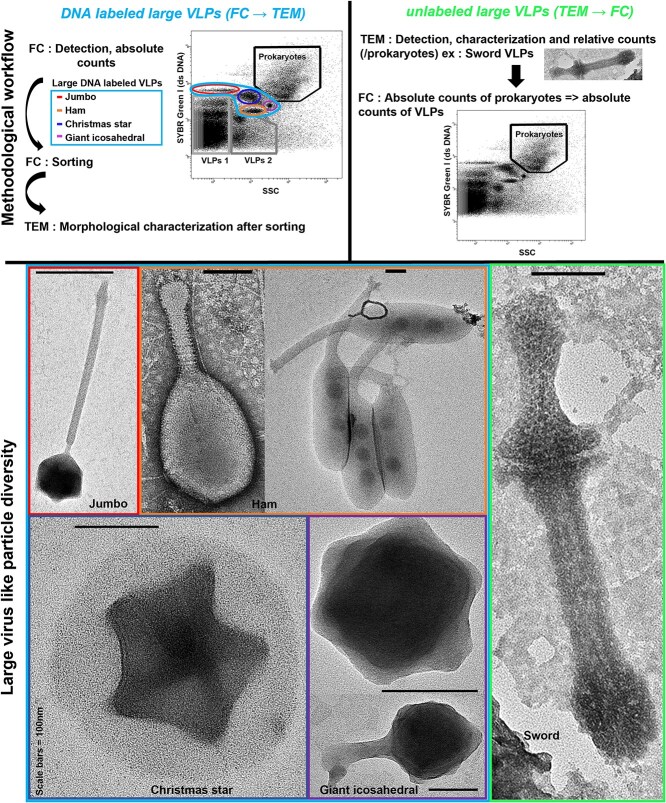

## Introduction

Viruses are major actors in the environment, affecting microbial community structure and biogeochemical cycles [1–4]. Viruses forming large particles (above 0.2 μm) that infect unicellular algae [5] or bacteria (“jumbo” phages [6]) have been known for several decades. However, they only came to prominence following the discovery of so-called “giant” viruses (up to 1.5 μm), including the iconic mimiviruses and pandoraviruses. These giant viruses, obtained mainly by the cultivation of *Acanthamoeba* sp. or algal species [7–10], have had a dramatic impact on our understanding of the evolution of viruses and blurred the boundaries between viruses and cells in terms of their physical dimensions and genomic complexity.

These culture-based studies enabled the detailed genotypic–phenotypic and biological characterization of isolated virus–host pairs [5, 6, 11, 12]. However, the isolation of viruses is hampered by our limited ability to cultivate most microbes such as heterotrophic microeukaryotes [13], l*et al*one their viruses.

Metagenomics has allowed us to progressively uncover many new giant virus genomes, demonstrating that these viruses are present and diverse in most ecosystems [2, 14–18]. Metagenome-assembled genomes (MAGs) can ascertain their genomic diversity, clarify their taxonomy [19, 20], and highlight their role in the evolution of cellular life forms [21–23]. For example, tens of MAGs were shown to form a diversified and prevalent group of viruses, representing a potential new phylum known as *Mirusviricota* related to the herpesviruses [24]. However, MAGs of giant viruses are often incomplete, containing little or no information about important biological and ecological features such as the host range, mode of infection, absolute abundance, or structure and composition of the viral particle. Metatranscriptomics can provide additional information by pointing to the host of giant uncultured viruses or accessing their activity within these hosts [25]. However, as with metagenomics, this approach does not provide access to the structure and composition of viral particles or to demonstrate their absolute abundances.

To tackle the limitations of both culture and omics-based studies, Fischer *et al*. [26] recently used transmission electron microscopy (TEM) to complement metagenomic approaches, thus revealing the surprising structural diversity of giant virus-like particles (VLPs) in forest soils. Although this methodology allows us to identify and categorize giant viruses based on morphological criteria, it is time-consuming and difficult to apply at high throughput. Consequently, this methodology alone is not well suited to tracking viral dynamics, thus preventing a better comprehension of the functional role played by viruses in natural ecosystems.

Flow cytometry (FC) is a rapid and inexpensive method that can be used to characterize environmental samples, leading to the absolute enumeration of nano- and microparticles. FC proved to be highly valuable for monitoring not only known viruses, mainly algal [27–30], but also viruses grouped into cytometric populations characterized by specific diffusion and fluorescence signals [31–33]. However, at the current stage and in the absence of a specific marker for large viruses, the composition of these cytometric populations in environmental samples remains uncertain.

The recently developed FC sorting of viruses offers new perspectives to fill the gap between viral characterization of a given population and ecological significance, as it can provide absolute counts of characterized viruses after sorting [34]. Nevertheless, to date, the application of FC sorting has remained limited to cultivable viruses [34] or genomic characterizations [35–38].

Applied alone or in combination with genomics, FC sorting does not provide information on the phenotypic diversity of viruses and consequently on the abundance of a given morphotype in complex environmental conditions. These shortcomings result in our inability to detect populations of unknown large viruses and to estimate their dynamics and ecology.

In order to fill the gaps associated with TEM and FC techniques used alone, we have developed a strategy coupling TEM with FC. These methodological developments allowed us (i) to characterize a considerable diversity of large VLPs in three French lakes, including several new types that probably infect unicellular eukaryotes and (ii) to provide insights into the dynamics and ecology of their populations.

## Materials and methods

### Study sites and sample collection

Samples were collected at the surface of three artificial freshwater eutrophic lakes: Fargette (45°44′39″N; 3°27′21″E, 86 dates from 2020 to 2024), Saint Gervais d’Auvergne (SG) (46°02′15″N; 2°48′43″E, 104 dates from 2020 to 2024), and Chambon (45°50′22″N; 3°30′17″E, 62 dates from 2022 to 2024). These lakes are located within a 120 km radius in the French Massif Central. Fixed samples with 1% (v/v) formaldehyde intended for the counts and determination of VLPs, prokaryotes (FC, TEM), and autotrophic/heterotrophic eukaryote communities (by light microscopy) were stored at 4°C until analysis. Unfixed samples intended for the analysis of autotrophic eukaryote communities by FC and for diversity analysis were stored at 4°C until processing within 4 h following sampling.

Marine samples were collected from North Atlantic waters during the APERO expedition onboard *Pourquoi Pas?* in June and July 2023 at three stations (48°27 167 N; 22°30 059 W, 50°37 250 N; 19°7098 W, 47°49 846 N; 15°46 690 W). The water was prefiltered through 1.2 μm glass-fiber filters (GFC Whatman) and concentrated 9- to 10-fold by ultrafiltration using a 0.2 μm cartridge (PES Vivaflow, Sartorius). An 8 ml aliquot of the concentrate was fixed with Electron Microscopy-grade glutaraldehyde (2% final concentration), flash-frozen, and stored at −80°C until analysis.

Additional details on study sites and sample collection are given in supplementary notes.

### Detection, counting, and characterization of large virus-like particles

#### TEM coupling with FC in both directions to detect, count and characterize large virus-like particles

**Figure 1:**
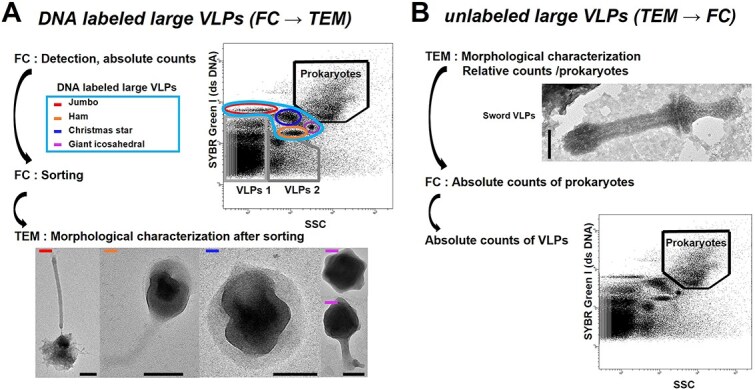
Workflow of the methodological strategy of coupling flow cytometry (FC)/transmission electron microscopy (TEM) to detect, characterize, and count large virus-like particles (VLPs) DNA-labeled (A) and unlabeled (B). (A) Workflow strategy to detect, enumerate, and characterize large DNA-labeled VLPs (FC first). FC provides detection and absolute counts of remarkable populations of large DNA-labeled VLPs. FC sorting then isolates these populations for TEM characterization. At the top, a dot plot of the gating strategy for the analysis of viral and microbial communities used in the temporal survey with emphasis on large DNA-labeled VLPs determined according to their DNA content (SYBR Green I) and side scatter (SSC) intensities. Seven populations were considered: Jumbo, Christmas star, Ham, giant icosahedral VLPs (the sum of these VLPs constitutes the large DNA-labeled VLPs), VLPs 1 and 2, and prokaryotes. At the bottom, negative staining electron micrographs of large DNA-labeled VLPs sorted by FC into FC-identified gates. (B) Workflow strategy to detect, enumerate, and characterize large DNA-unlabeled VLPs (TEM first). TEM provides detection and relative counts (relative to prokaryotes) of large remarkable viruses. FC analysis provides the absolute counts of prokaryotes, allowing the absolute abundances of particles detected in TEM to be obtained. At the top, negative staining electron micrographs of a large DNA-unlabeled VLP detected in environmental samples. At the bottom, a dot plot of the gating strategy for the analysis of prokaryotes used to convert relative counts of unlabeled large VLPs into absolute counts. Scale bars = 100 nm.

TEM and FC coupling can be applied by first using TEM or FC. The strategy depends on our ability to detect VLPs based on their labeling with the DNA SYBR Green I (SGI) dye (S7585, Invitrogen, Thermo Fisher Scientific). Using FC first (FC → TEM, Fig. 1A) can only be used to detect and count large VLPs emerging from a specific population based on their labeling intensity. Counts are applied directly to the cytogram. With this methodology, regular sorting and TEM characterization are required to assess population specificity. Here, we can apply this FC strategy first to jumbo phages, Ham, Christmas star [26] (CS), and giant icosahedral VLPs (GIVs). The TEM first method (TEM → FC, Fig. 1B) can be used for all labeled and unlabeled VLPs. This TEM’s first strategy is the only one that can be used to detect and count unlabeled particles, in our case Sword VLPs. These particles are first detected in TEM, and a count relative to prokaryotes is obtained by TEM. This relative count is then converted to an absolute count using FC counting of prokaryotes. The methodologies associated with these methods are detailed in the following sections. The VLPs’ names are used to refer to their original shapes (Ham, Sword) or are derived from a previous identification (CS [26]).

### Quantification of labeled large DNA virus-like particles and prokaryotes

Counts of DNA VLPs and prokaryotes from fixed samples were performed in triplicate by FC using the DNA dye SGI according to Brussaard [29] with a BD FACSAria Fusion SORP (BD Biosciences, San Jose, CA, USA) equipped with an air-cooled laser delivering 50 mW at 488 nm with 502 longpass, and a 530/30 bandpass filter setup. All cytometric data were acquired and analyzed with BD FACSDiva 9.0 software. The gating and counting strategy are provided in Fig. 1A. Dates with gate overlaps, notably from prokaryotes or debris populations, were excluded from the analysis. VLP1 is dominated by small bacteriophages (<200 nm) [39, 40] and small eukaryotic algal viruses [41–44]. VLP2 is mainly composed of a tailless icosahedral virus of around 100 nm in diameter [28, 42, 45]. We identified populations of large DNA VLPs characterized by high side scatter (SSC) and/or high SGI signals between prokaryote and small VLP1–2 populations.

### Fluorescence-activated large virus-like particle sorting

For VLP sorting, we selected dates with remarkably large VLP populations: Fargette on 1 March 2022, SG on 14 February 2022, and Chambon on 16 December 2022, and 12 January 2024.

VLP sorting was performed with the FACSAria Fusion SORP (BD Biosciences) flow cytometer using a 488 nm argon laser for excitation using the “single cell” sort mode. Sorting instruments and reagents were decontaminated as recommended by the manufacturer (Prepare for Aseptic Sort, BD Biosciences). The threshold of the cytometer was triggered at the minimum in the green fluorescence channel, and the sorting gates were based on SGI fluorescence and SSC.

From each of the distinct populations mentioned above, between 3.10^5^ and 3.10^6^ VLPs were sorted for TEM identification. For this sorting, we used sterile NaCL (2.24 g.l^−1^) diluted in ultrapure water solution as sheath fluid. This NaCL concentration was determined to permit correct deflection (concentration range test, data not shown) in order to prevent salt contamination of the TEM observations.

### Quantification of specific large virus-like particles with or without flow cytometry signal and morphological characterization

VLPs and microbial communities from fixed samples were collected by centrifugation at 20 000 x *g* for 20 min at 14°C directly on 400-mesh electron microscopy copper grids covered with carbon-coated Formvar film (AO3X, Pelanne Instruments, Toulouse, France). Particles were over-contrasted using uranyl salts. Specific viruses were detected, characterized, and counted by TEM using a Jeol JEM 2100-Plus microscope (JEOL, Akishima, Tokyo, Japan) equipped with a Gatan Rio 9 CMOS camera (Gatan Inc., Pleasanton, USA) operating at 80 kV and ×50 000 to ×150 000 magnifications. TEM images were acquired and measurements made with DigitalMicrograph-GMS 3 (Gatan Inc., Pleasanton, USA). Resizing as well as light and contrast corrections were carried out with ImageJ [46] or Photos Microsoft (Microsoft Corporation, Redmond, Washington, USA). Scale bars were retraced and formatted manually.

The number of specific VLPs with (Ham, CS) or without FC signals (Sword) resulted from the multiplication of the VLP/prokaryote ratio determined using TEM by the prokaryote concentration obtained using FC (Fig. 1B).

The terms “large,” “jumbo,” or “giant” VLPs were used in relation to (i) the size of VLPs (length > 200 nm on one of their axes), which present either an icosahedral or a head–tail–fiber morphology, (ii) FC characteristics (high values of SGI (DNA dye) or SSC (complexity/size) mean fluorescence intensities), and (iii) the visual identification (TEM) of lytic events of putative hosts.

For VLPs presenting an identified cytometric population (CS, Ham), we validated the reciprocity between the methods FC → TEM (Fig. 1A) and TEM → FC (Fig. 1B) by their positive correlation (Supplementary Fig. S1). In the analysis of their dynamics, we present the results of the method with the maximum number of points (i.e. FC → TEM for CS VLPs and TEM → FC for Ham VLPs). GIVs were only counted by FC. For VLPs with no detectable cytometric signature using SGI (Sword), the TEM → FC approach was the only method for detecting them.

Large DNA-labeled VLP populations (CS, Ham, GIVs, and jumbo-like phages) and Sword VLPs (large DNA unlabeled VLPs) were combined to consider the total community of large VLPs. Jumbo-like phages were removed to consider the potential population of presumed large microeukaryote VLPs.

### Biotic parameter analysis

Autotrophic microbial communities were analysed in situ using a spectrofluorometric probe (green algae, Cyanobacteria, Cryptophyta) (BBE FluoroProbe, Moldaenke GmbH, DE) or in the laboratory by FC using a BD LSR Fortessa X-20 (BD Biosciences). Heterotrophic microeukaryotes were counted by light microscopy using the Utermöhl method. For a selection of samples, the diversity of the microbial communities was analyzed by metabarcoding. Briefly, samples were collected on a 0.2 μm polycarbonate filter (Millipore); then, gDNA was extracted, and the V4 region of the eukaryotic SSU RNA gene was amplified by PCR using TAReuk454FWD1 (5′-CCAGCASCYGCGGTAATTCC-3′) and TAReukREV3 (5′- ACTTTCGTTCTTGATYRA-3′) adaptor-labeled primers [47] and was sequenced by Illumina MiSeq, v3, 2*300 cycles.

A detailed description of the techniques used to analyze biotic parameters is provided in supplementary notes.

### Data analysis

All statistical analyses were performed using R software (version 4.1.3, R Foundation for Statistical Computing, Vienna, Austria). Potential relationships among all variables were tested by pairwise correlations (Spearman correlation analyses) using the “rcorr” function from the “Hmisc” package (v. 5.2-0 10.32614/CRAN.package.Hmisc).

## Results and discussion

As with jumbo phages, the diversity and ecology of large aquatic VLPs associated with microeukaryotes remains largely underexplored. Here, we report the discovery of large and abundant VLPs with morphotypes never seen before, e.g. the VLPs named Ham and Sword because of their original shapes. While the VLPs described as “Christmas stars” [26] have so far only been detected in soils, we also report their detection in fresh and marine waters. Finally, we present the morphological characterization and tracking of abundant GIVs and suggest the ecological importance of the entire community of large VLPs presumably associated with microeukaryotes.

### Large virus-like particles: abundant and dynamic players in viral ecology

Data from cultures [5, 6, 10–12], microscopic observations [26], and genomic analyses [2, 14–17] have led to fundamental discoveries in the study of large viruses. However, in the absence of a methodology to detect, characterize, and quantify their noncultivable representatives, their diversity and ecological importance remain underexplored. To fill this knowledge gap, we coupled FC and TEM (Fig. 1) to study large VLPs in aquatic ecosystems. FC not only allowed the detection and absolute counts of large VLPs [29] carrying DNA genomes fluorescently labeled with SGI but also enabled the comparison of corresponding subpopulations on the basis of fluorescence signals associated with SSC and DNA fluorescence [28, 31, 34] (Fig. 1A). TEM allowed us to assess the homogeneity and morphological characteristics of the populations sorted using FC, as well as the visualization and quantification of large VLPs (Fig. 1A and B) from environmental samples that were not labeled in the FC analysis.

Using this protocol, the populations of large VLPs were morphologically characterized and quantified in samples collected over successive years (2020–24) in three eutrophic freshwater lakes [Fargette, Saint Gervais d’Auvergne (SG), and Chambon]. Four populations forming a group of large DNA-labeled VLPs were identified on cytograms: jumbo-like phage, CS, Ham, and GIVs. We were unable to identify the cytometric population corresponding to Sword VLPs after FC sorting; these are mentioned as large DNA unlabeled VLPs.

**Figure 2:**
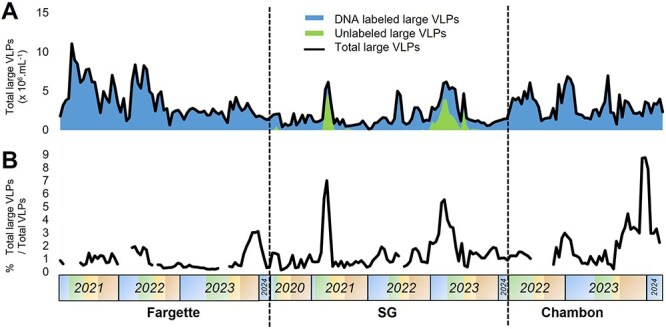
Seasonal dynamics of the abundance (A) and of the relative abundance (B) of total community of large virus-like particles (VLPs) (DNA-labeled, i.e. the sum of jumbo phage, Christmas star, Ham, and Giant icosahedral VLPs, and unlabeled, i.e. sword VLPs), in lakes Fargette, SG, and Chambon. Each data represents the average of triplicates, *n* = 209.

Large VLPs were consistently present in the studied systems, ranging from 0.02 to 1.1 × 10^7^ VLPs.ml^−1^ (Fig. 2A), i.e. from 0.2% to 8.8% of total VLPs (Fig. 2B). Their dynamics were characterized by phases of production in spring or late autumn alternating with low detection phases (Fig. 2). Notably, among the large VLPs, at a specific time up to 77% were unlabeled. To decipher the significance of aquatic large VLPs, we explored their morphological diversity and ecological patterns (i.e. dynamic, interactions with microbial communities) while separating the VLPs into two groups, namely, large VLPs likely associated with microeukaryotes, detailed in the following section, and jumbo phages, detailed in the supplementary results.

### Large virus-like particles likely associated with microeukaryotes: amazing diversity and unexpected ecological significance

#### Ham virus-like particles: atypical morphology of a new viral type

**Figure 3:**
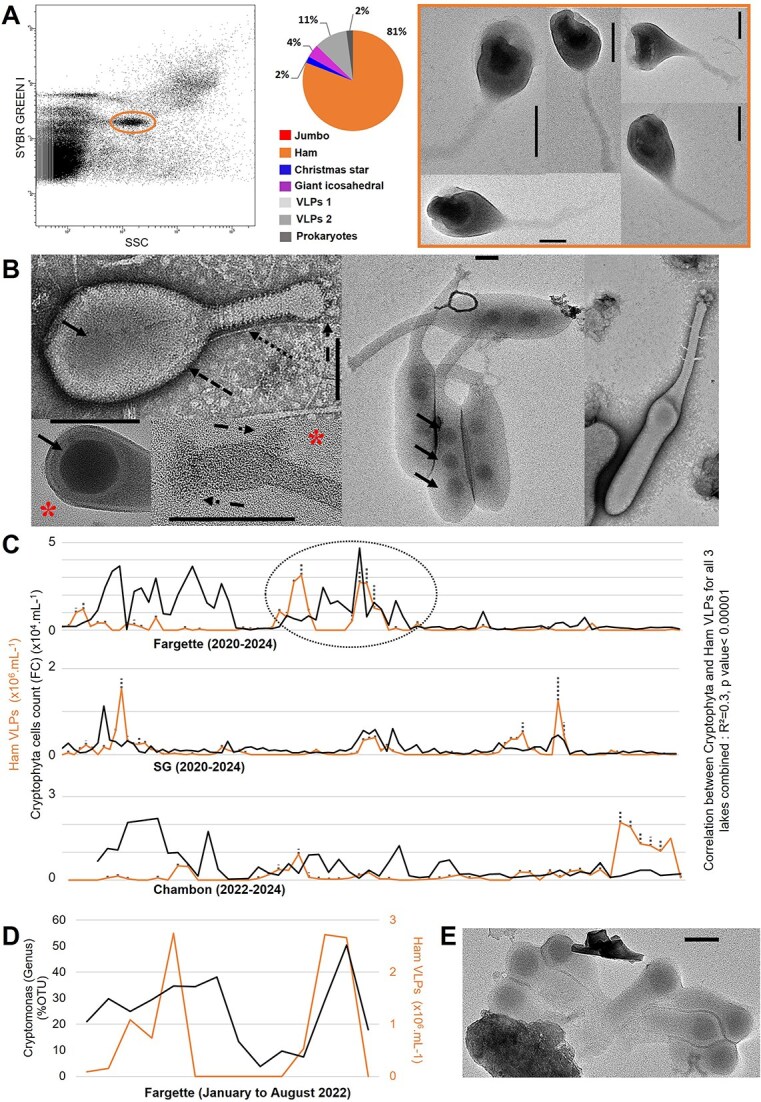
Detection and morphological and ecological characterization of Ham virus-like particles (VLPs) detected in eutrophic French lakes. (A) Flow cytometry (FC) detection of remarkable population of Ham VLPs (circle in the cytometric profile) and pie chart of the diversity of entities recorded by transmission electron microscopy after FC sorting in the corresponding population with micrographs of the Ham VLPs sorted. Note the deleterious effect of sorting with visible damage on the capsid of Ham VLPs. (B) Negative staining electron micrographs of Ham VLPs in which we detected one, two, or three icosahedral structures (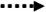) contained within a surrounding structure with a head (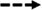)–tail (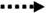) morphology. 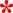
Illustrated zoom on head or tail. Note the spiral-shaped molecular structure around the tail (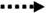) and the presence of fibers (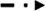) at the end of the tail. (C) Seasonal dynamics of the abundance of Ham VLPs and Cryptophyta cells, in lakes Fargette, SG, and Chambon. Each data represent the average of triplicates; dotted lines indicate standard deviation. *n* = 252. (D) Focus on remarkable infection periods on the covariations of Ham VLPs and *Cryptomonas* sp*.* (% OTU) in Lake Fargette from January to March 2022. (E) Negative staining electron micrographs of Ham VLPs derived from a lytic event. Scale bars A, B, E = 100 nm.

During our study, we detected a particular cytometric signature corresponding to a new type of VLPs, which we named Ham. The average fluorescence intensities derived from the DNA labeling and SSC of these particles were between VLPs2 and prokaryotes, suggesting that they have large genomes (Figs 1A and 3A).

The Ham VLPs are polymorphic particles with lengths ranging from 450 to 1500 nm (Fig. 3). They are characterized by an ovoid head ranging from 240 to 900 nm in length and from 135 to 200 nm in width, a tail surrounded by an apparently helical assembly ranging from 140 to 600 nm in length and from 40 to 50 nm in width. The end of the tail is decorated with fibers. Some morphotypes have a bulge at the end of the tail. A notable feature is the presence of an icosahedral structure of 75–80 nm inside the head. Two or three icosahedrons inside the largest form were occasionally observed (Fig. 3B). These multi-icosahedron forms were rare (<1%), except in Lake Chambon from 20 December 2023 to 10 January 2024, in which they could represent up to 64% of the total Ham VLPs counted. To our knowledge, this is the first description of a head–tail VLP with an icosahedral structure covered by an external layer. Morphologically, the closest relative appears to be the enigmatic virions of Meelsvirus [48].

Ham VLPs were encountered in all three lakes considered in this study and reached 3.1 × 10^6^ VLPs.ml^−1^ with a sudden and massive development strategy (Fig. 3C). The proliferation dynamics of Ham VLPs strongly correlated with the Cryptophyta population (*R*^2^ = 0.3, *P*-value <.00001). Metabarcoding data showed that at the time of the VLP bloom, eukaryotic communities were dominated by Cryptophyceae, suggesting that Ham VLPs co-evolved with Cryptophyceae species. We are focusing in particular on the potential “prey–predator” relationship between *Cryptomonas* sp. and Ham VLPs (Fig. 3D). This model of interaction has been frequently observed for large algal viruses [33, 49–53]. Although host specificity, determined here empirically, must be verified, these observations suggest that Ham VLPs infect and control autotrophic microeukaryotes, presumably *Cryptomonas* sp.

The pleomorphism of Ham VLPs suggests that it could be represented by different phylotypes, a possibility reinforced by the ubiquity of Ham VLPs and their development at different times of the year under contrasting environmental conditions (temperature range 5°C–20°C). However, its development strategy and malleable morphology could also support the hypothesis of specific polymorphic VLPs.

#### Christmas star virus-like particles: widespread common large viruses of eukaryotes?

**Figure 4:**
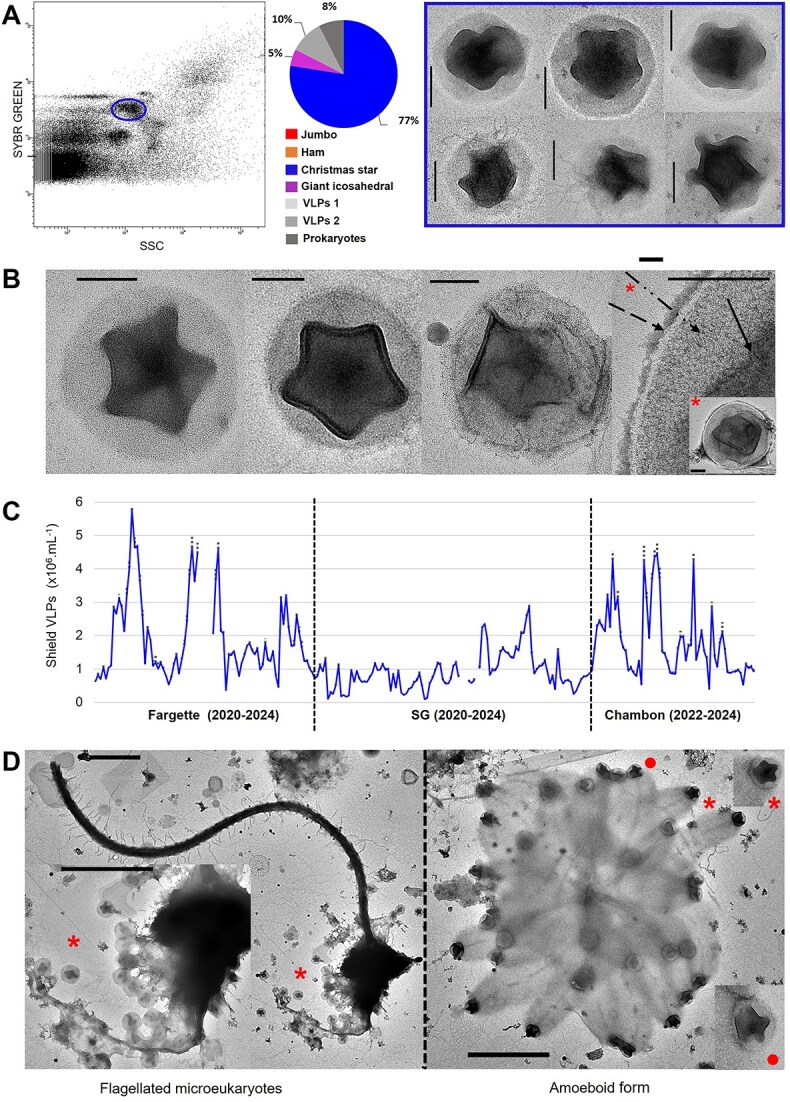
Detection, morphological and ecological characterization of Christmas star (CS) virus-like particle (VLPs) detected in eutrophic French lakes. (A) Flow cytometry (FC) detection of a remarkable population of CS VLPs (circle in the cytometric profile) and a pie chart of the diversity of entities recorded by transmission electron microscopy after FC sorting in the corresponding population with negative staining electron micrographs of the CS VLPs sorted. (B) Negative staining electron micrographs of CS VLPs in which we identified an inner pentagonal structure (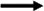) surrounded by a tegument (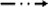) and an envelope (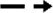)-like structure. Note the stargate-like motif in the center of the inner structure. (C) Seasonal dynamics of the abundance of CSVLPs, in lakes Fargette, SG, and Chambon. Each data represent the average of triplicates; dotted lines indicate standard deviation. *n* = 243. (D) Negative staining electron micrographs of CS VLPs derived from lytic events of a flagellated microeukaryote (left) or an amoeboid form host (right). Scale bars A, B = 100 nm, D = 1 μm. *Illustrated zoom parts.

Like Ham, CS VLPs exhibited a specific cytometric signature with fluorescence intensities in the range of small prokaryotes and slightly lower than jumbo-like phages. The cytometric signatures of Ham and CS VLPs correspond to those of large genome DNA viruses [27–31], suggesting a large genome (Figs 1A and 4A).

CS VLPs have an electron-dense inner capsid (140–300 nm in diameter) surrounded by a less electron-dense spherical outer structure (194–460 nm in diameter) (Fig. 4A and B, Supplementary Fig. S2A). The inner capsid features a pentagonal planar projection with a 5–9 nm thick border. Some representatives of CS VLPs suggest that the external structure is composed of a tegument and an envelope, as is characteristic of viruses in the order *Herpesvirales* (Fig. 4B). However, the nature of this envelope-like structure is unclear, as it could be lipidic, as in herpesviruses, or based on glycans, as in *Megavirinae* [54, 55]. Unlike *Megavirinae*, the hairy appearance resulting from the arrangement of fibrils has not been clearly observed, and some CS representatives listed here are smaller than *Megavirinae*. However, one example of a particular *Megavirinae,* namely, Cotonvirus (400 nm in capsid diameter), shows that the density of fibrils on the capsid can have a smooth appearance under TEM [56].

We observed cells from different types infected with CS VLPs: amoeboid forms 3 μm in diameter and different types of flagellated microeukaryotes with a head ranging from 1.8 to 6 μm with one or two flagella (Fig. 4D, Supplementary Fig. S2B). The nature of their hosts and their trophic mode remain to be determined. However, some of them did not exhibit or lacked pigment content under fluorescence microscopy. This putative broad host range was also suggested by positive correlations between CS VLPs and almost all the microbial compartments mentioned above (*P*-value <.01). We assumed that CS VLPs can infect flagellated heterotroph microeukaryotes (FHMs). *Cafeteria roenbergensis* virus [57] and Klosneuviruses [58, 59] are rare viruses morphologically described capable of infecting FHM [60]. CS VLPs are ubiquitous and were encountered in all three lakes studied here. We quantified CS VLPs with remarkable abundances reaching 13.7 × 10^6^ VLPs.ml^−1^ and with a dynamic characterized by irregular phases of development (Fig. 4C). Coupled to a mean BS observed of 27 (Fig. 4D, Supplementary Fig. S2B), these results suggest strong control of their host populations.

Their ubiquitous distribution was reinforced by our observations of numerous CS morphotypes in marine systems in which they can reach 8.3 × 10^4^ VLPs.ml^−1^ in surface area (Supplementary Fig. S2C) as well as by previous observation in soils [26]. Coupled with the pleomorphism of CS VLPs and their potential host range, we suggest that the phylum to which they belong is very diverse and plays a major ecological role in the regulation of microeukaryotes in many environments. The morphology of these viruses could be a common feature shared by many types of unicellular eukaryotic viruses.

Finally, we also recorded strikingly rare forms of CS-like VLPs (<1% of our observations) bearing tails or fibrils or in tandem (Supplementary Fig. S3), up to 600 nm in diameter. We cannot certify the presence of these tail forms in the cytometric gate corresponding to the CS VLPs.

#### Giant icosahedral virus-like particles: novelties in common large viruses

**Figure 5:**
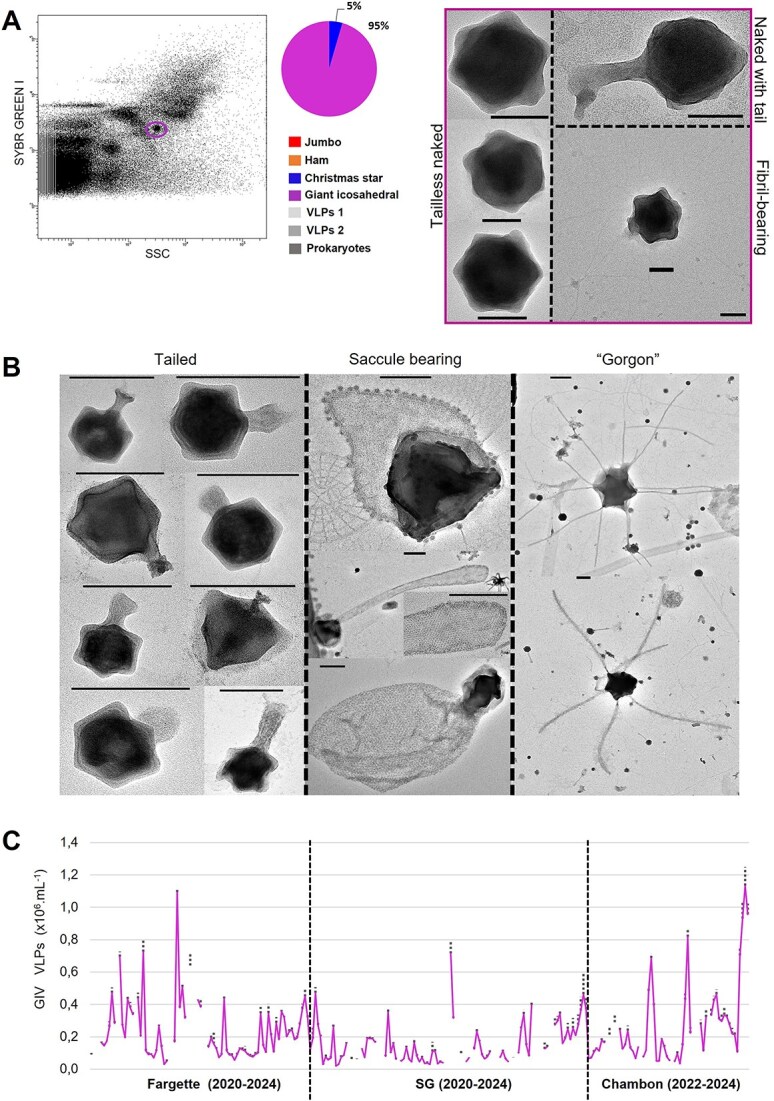
Detection and morphological and ecological characterization of giant icosahedral virus-like particles (GIVs) detected in eutrophic French lakes. (A) Flow cytometry (FC) detection of a remarkable population of GIV (purple circle) and a pie chart of the diversity of entities recorded by transmission electron microscopy after FC sorting in the corresponding population with micrographs of the GIV sorted. (B) Negative staining electron micrographs of various GIV, tailed, bearing a saccule or “gorgon.” Scale bars A = 100 nm, B = 200 nm. (C) Seasonal dynamics of the abundance of GIVs, in lakes Fargette, SG, and Chambon. Each data represent the average of triplicates; dotted lines indicate standard deviation. *n* = 209.

In our study, we detected an FC population corresponding to GIVs (Fig. 5A). Their cytometric signature associated with DNA and SSC suggested a large genome and high internal complexity, respectively (Figs 1A and 5A).

In this population, three morphotypes were observed: (i) tailless naked giant icosahedron (79% of the population in Chambon on 10 January 2024) of 212 nm in diameter; (ii) tail giant icosahedron (20%) of 180 nm in diameter with a tail of 150 nm length; and (iii) fibrils bearing giant icosahedron (<1%) of 230 nm in diameter (Fig. 5A). The morphologies and sizes of the first two groups are similar to those observed for members of the *Megaviricetes* class, whereas no affiliation was proposed for the observed tailed types. Illustrations of various-tailed GIVs are provided in Fig. 5B. Finally, we also reported rare (<1% of the GIV observations) GIVs with a saccule (up to 446 nm icosahedral diameter) and morphotypes with tubular appendages (Fig. 5B) previously recorded in forest soils as “Gorgon” [26]. We cannot certify the presence of these amazing tailed forms in the cytometric gate corresponding to the GIVs.

Abundances of GIVs could reach 1.1 × 10^6^ VLPs.ml^−1^ and showed a dynamic with an irregular phase of development (Fig. 5C), suggesting a strong control of their host population. GIVs were recorded year-round, suggesting that they were dominant with numerous phylotypes and had a potential broad host range, as supported by their correlations with almost all microbial compartments (*P*-value <.01).

Our data are among the first to document the absolute abundance of this presumably diverse population of *Megaviricetes*. The limited data available to date stemmed from specific isolated and fully described marine phytoplankton viruses during an algal bloom [49–51, 61, 62].

#### Sword virus-like particles: new dynamic and specific viruses of microeukaryotes?

**Figure 6:**
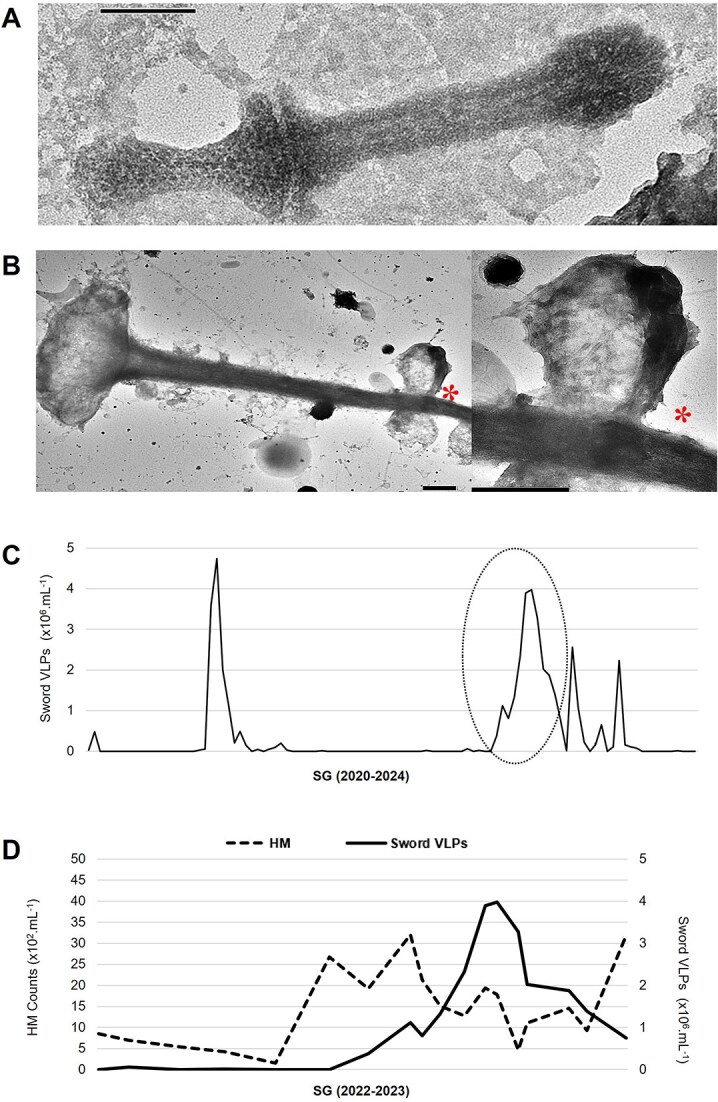
Detection and morphological and ecological characterization of Sword virus-like particles (VLPs) detected in eutrophic French lakes. (A) Negative staining electron micrographs of Sword VLPs with a head (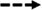)–tail (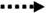) morphology terminated by fibers (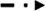). (B) Negative staining electron micrographs of Sword VLPs derived from a lytic event in a microeukaryote host. Some Sword VLPs in 7B are broken or are not fully assembled, i.e. only showing the tail. *Illustrated zoom part. Scale bars A = 100 nm, B = 1 μm. C, seasonal dynamics of the abundance of Sword VLPs, in lakes Fargette, SG, and Chambon. Each data represent the average of triplicates; dotted lines indicate standard deviation. *n* = 252. (D) Focus on remarkable infection periods on the covariations of sword VLPs and heterotrophic microeukaryotes (HMs) in lake SG from 18 October 2022 to 12 April 2023.

We report on the discovery of original VLPs above 200 nm, which we called Sword VLP on account of their shape (Fig. 6). One particularity was that they are undetectable by FC based on the measured parameters. This lack of FC detection using DNA dye raises the question about the nature of their nucleic acid, probably non-DNA. Another explanation is that the signal is hidden within the bulk of VLPs1, VLPs2, or prokaryotes. However, despite their high abundances during the infection period, we found no correlation between TEM counts and any viral cytometric population.

Sword VLPs are a head–tail–fiber VLP measuring 635 nm in length (Fig. 6A). This viral type is characterized by an elongated dumbbell-shaped head measuring 220 nm in length and between 45 and 135 nm in width, with a sheath-like tail of 370 nm in length and 60 nm in width and fibers distributed at the end of the tail. A thin 9.5 nm thick groove separates the presumed head from the tail. The head has a distinctive substructure resulting from a patterned arrangement of 6 nm diameter subunits. Morphologically, the closest relative appears to be a filamentous VLP previously imaged in a freshwater lake [63]. We reported various observations of numerous Sword VLPs inside or around a heterotrophic microeukaryote possessing a sheath-like structure at least 35 μm long with a funnel-shaped end (Fig. 6B, Supplementary Fig. S4). We are unable to specify the host bearing this structure. Several hypotheses have emerged: a ciliate lorica or a fungi mycellar or a hyphoid structure. The BS observed for Sword VLPs appeared to be around 115. We found Sword VLP only in Lake SG. Their dynamic, reaching 4.7 × 10^6^ VLPs.ml^−1^, was characterized by a sudden and massive development strategy (Fig. 6C).

On an expanded spatiotemporal scale, we were unable to identify significant correlations (*P*-value >.01) between Sword VLPs and the autotrophic microbial compartments considered in this study. Nevertheless, focusing on the remarkable viral infection peaks from 18 October 2022 to 12 April 2023, we showed a clear interaction in the “prey–predator” model between Sword VLPs and heterotrophic microeukaryotes (Fig. 6D). These dynamic analyses support the TEM observations showing a putative heterotrophic microeukaryote host for Sword VLPs and suggest a high involvement of Sword VLPs in the demise of their host population.

Finally, the reproducibility of morphological observations of the Sword VLPs and their host, coupled with their development strategy, suggests that they are highly specialized VLPs.

#### Ecological significance of presumed large microeukaryote virus-like particles

This study represents one of the first time series of large VLPs presumably associated with microeukaryotes and provides novel insights into their numerical significance in aquatic systems. Detection limitations such as the inability to detect Sword VLPs using FC will likely lead to an underestimation of the total number of these VLPs. Nevertheless, our data emphasize their numerical importance in freshwater systems, with total abundances ranging from 0.2 to 6.2 × 10^6^ VLPs.ml^−1^. As such, large microeukaryote VLPs accounted for between 0.1% and 2.7%, 0.1% and 6.8%, and 0.3% and 7.8% of the total viral stock in Lakes Fargette, SG, and Chambon, respectively (Supplementary Fig. S5). These variable contributions point to the alternating control of microbial systems by large microeukaryote VLPs versus smaller viruses (mostly phages). Of notable interest, this study also reports the significant contribution of large VLPs in an offshore marine site and particularly CS VLPs, thus expanding the distribution of this morphotype to all aquatic systems.

On an annual basis, the productive periods of large VLPs occurred mainly in spring, with some exceptions such as the summer peak in 2022 in Lake Fargette. This strong temporal dynamism was also underlined by the rapid and unexpected successions of specific large VLPs. Some morphotypes (Ham, CS, Sword) can evolve from undetectable levels to over 77% of the total large VLPs in a matter of days. At other times, a mixed community of large VLPs was observed in equal proportions (Supplementary Fig. S5A). Our results were consistent with the “Bank model” [64], which suggests that only a small fraction of the viral community is active and abundant at any given time, while most populations are rare and dormant, forming a seed bank that can “Kill-the-Winner” when hosts reach critical abundance thresholds [65]. It is worth noting that naked GIVs were only dominant once in Lake SG (55% of the total large VLP community on 30 May 2022) and that they represent only 12% of all the combined data on average. Thus, large VLPs not previously considered morphologically (Ham, CS, Sword) could be prevalent in the microeukaryote ecology of the aquatic environment.

To decipher the effect of large microeukaryote VLPs on host communities, we monitored the dynamics of their abundance concomitantly with those of microbial communities (abundance and diversity). We reported “prey–predator” patterns between autotrophic microeukaryotes and Ham VLPs and between heterotrophic microeukaryotes and Sword VLPs (Figs 3D and 6D). Both autotrophic and heterotrophic microeukaryotes can contributed significantly and variably to the stock of large microeukaryote VLPs. We observed no significant correlation with abiotic parameters (Supplementary Fig. S6), confirming the importance of the microbial environment in the control of large microeukaryote VLPs. The high values of maximum ratios between the total large microeukaryote VLPs and microeukaryotes (325, 949, and 508 in Lakes Fargette, SG, and Chambon, respectively) point to the significant potential of large microeukaryote VLPs to control microeukaryote populations.

The potential influence of large VLPs on microeukaryote succession (Supplementary Fig. S7) was assessed by analyzing the temporal dynamics of microeukaryotic diversity (Shannon index) in relation to the abundance of large VLPs of microeukaryotes (Supplementary Fig. S8). Our findings showed that episodes of VLP production, regardless of the dominant type, coincided with a decrease in eukaryote diversity. The effects of large VLPs may be cumulative, as in the example of the simultaneous development of Sword and CS VLPs in Lake SG, or they may follow each other successively, as in the example of the successive production of Ham and CS VLPs in Lake Fargette. Although it is clear that large VLP communities were an essential factor in regulating the succession of microeukaryote communities in our study sites, the lack of information regarding their life traits (e.g. infection mode, host specificities) or subcellular interactions limits our understanding of their overall impact. Dedicated efforts to isolate and characterize the interactions of large VLPs must be pursued in order to gain insights into the role of this underexplored yet significant component of the virosphere by means of cultures, subcellular observations, and transcriptomic analyses [3, 25, 66].

## Conclusions and perspectives

Thanks to the combination of FC and TEM, we shed light on the astonishing phenotypic diversity of large VLPs in aquatic ecosystems, with the identification of specific cytometric populations and novel morphotypes never previously described. These VLPs, which infect prokaryotes or microeukaryotes, undoubtedly play a role in the control of their host population. Each type of VLP probably represents different taxonomic levels and specializations (from specialist to generalist). Some notably seem to infect heterotrophic microeukaryotes, for which few viruses have been reported. The global community of large VLPs, considered here for the first time on a large temporal scale, showed marked dynamics in terms of both their abundance and diversity, with each of the studied types either succeeding or accumulating. We suggest that these viral communities have a very strong impact on ecosystem functioning by controlling the dynamics of prokaryotic and eukaryotic communities, both autotrophic and heterotrophic, and by controlling their diversity, which implies their major role, hitherto unimagined at such a scale, in the functioning of the ecosystem.

These discoveries impact our perception of the virosphere, its diversity, and the role played by large viruses in the dynamism of their hosts and the functioning of aquatic food webs. This study urges us to investigate the exact nature and genomic characterization of these VLPs and to identify their host to fully explore these concepts.

## Supplementary Material

Supplementary_figures_1-8_Billard_et_al_2025_ycaf098

Supplementary_Notes_Billard_et_al_2025_ycaf098

Supplementary_results_Billard_et_al_2025_ycaf098

## Data Availability

Relevant data supporting the key findings of this study are available in the article and the Supplementary Information file. The raw dataset underlying this article is available in ZENODO, at https://dx.doi.org/10.5281/zenodo.15574592 [67].
