# Supplementary material for: Unexpected diversity and ecological significance of uncultivable large virus-like particles in aquatic environments
Source: ISME Commun. 2025 Jun 5;5(1):ycaf098. doi: 10.1093/ismeco/ycaf098 (PMC12204322; doi:10.1093/ismeco/ycaf098)
Supplement: Supplementary_figures_1-8_Billard_et_al_2025_ycaf098 [file supplementary_figures_1-8_billard_et_al_2025_ycaf098.docx]

**Supplementary Figures**

**Unexpected diversity and ecological significance of uncultivable large virus-like particles in aquatic environments**

Hermine Billard, Maxime Fuster, François Enault, Jean-François Carrias, Léa Fargette, Margot Carrouée, Perrine Desmares, Tom O. Delmont, Pauline Nogaret, Estelle Bigeard, Gwenn Tanguy, Anne-Claire Baudoux, Urania Christaki, Télesphore Sime-Ngando, Jonathan Colombet*

Affiliations

Laboratoire Microorganismes : Génome et Environnement (LMGE), UMR CNRS 6023, Université Clermont-Auvergne, F-63000 Clermont-Ferrand, France

Hermine Billard, Maxime Fuster, François Enault, Jean-François Carrias, Léa Fargette, Margot Carrouée, Perrine Desmares, Télesphore Sime-Ngando, Jonathan Colombet

Génomique Métabolique, Genoscope, Institut François Jacob, CEA, CNRS, Univ. Evry, Université Paris-Saclay, Evry, France

Tom O. Delmont

Sorbonne Université, CNRS, Station Biologique de Roscoff, FR2424, Roscoff, France

Gwenn Tanguy

Sorbonne Université, CNRS, Station Biologique de Roscoff, UMR 7144, Roscoff, France

Estelle Bigeard, Pauline Nogaret, Anne-Claire Baudoux

UMR CNRS 8187 LOG, Université Littoral Côte d’Opale, Université de Lille, Wimereux, France

Urania Christaki

*Corresponding author: Jonathan Colombet.

Email : [jonathan.colombet@uca.fr](mailto:jonathan.colombet@uca.fr)

**Contents**

**Supplementary figure 1**

**Supplementary figure 2**

**Supplementary figure 3**

**Supplementary figure 4**

**Supplementary figure 5**

**Supplementary figure 6**

**Supplementary figure 7**

**Supplementary figure 8**


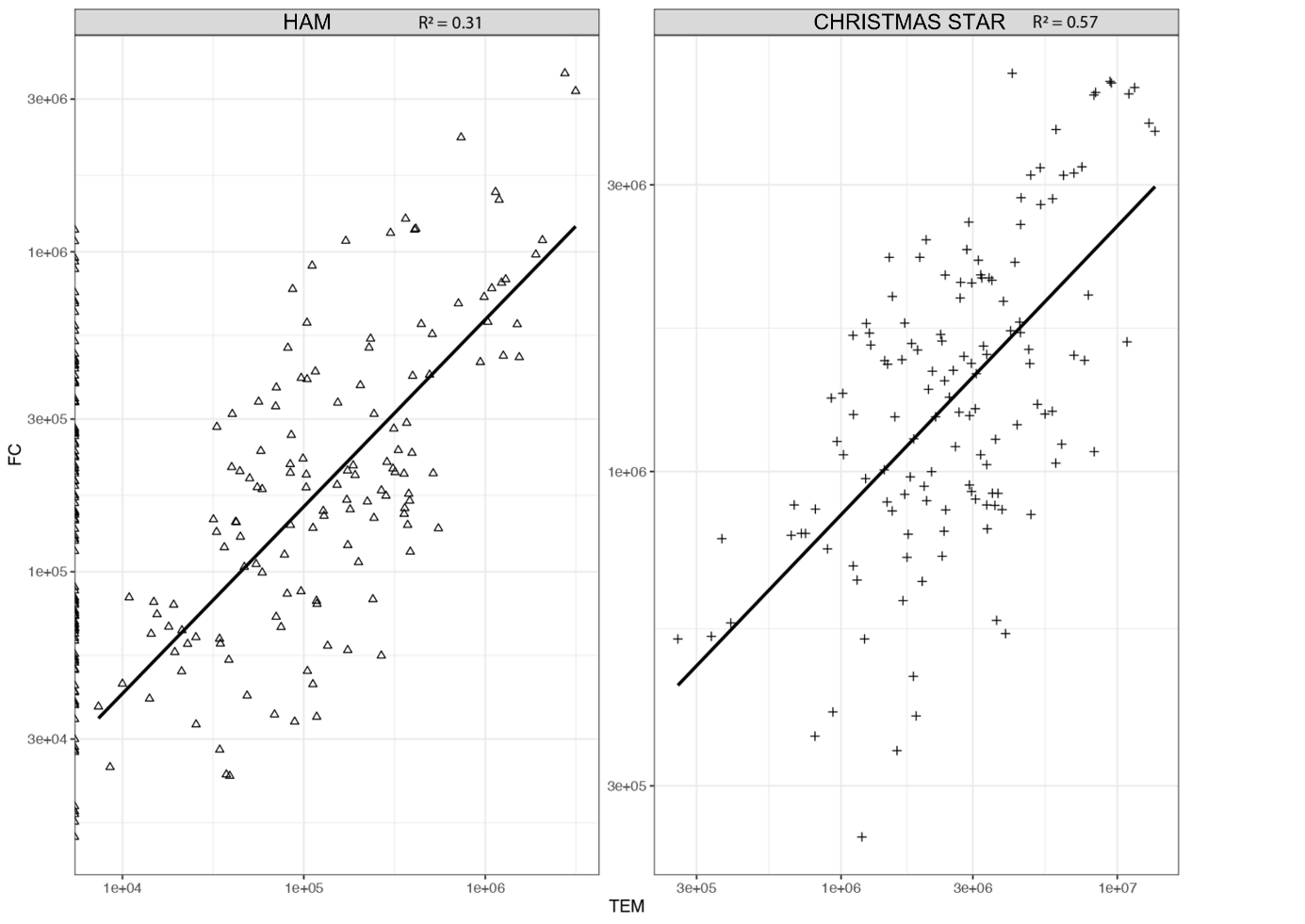


**Supplementary figure 1** Spearman correlative analysis (all data from three lakes combined) between counts of Christmas star (CS) or Ham virus-like particles by flow cytometry (FC) compared to counts by transmission electron microscopy (TEM). p values < 0.001. n Ham = 247, n CS = 135.


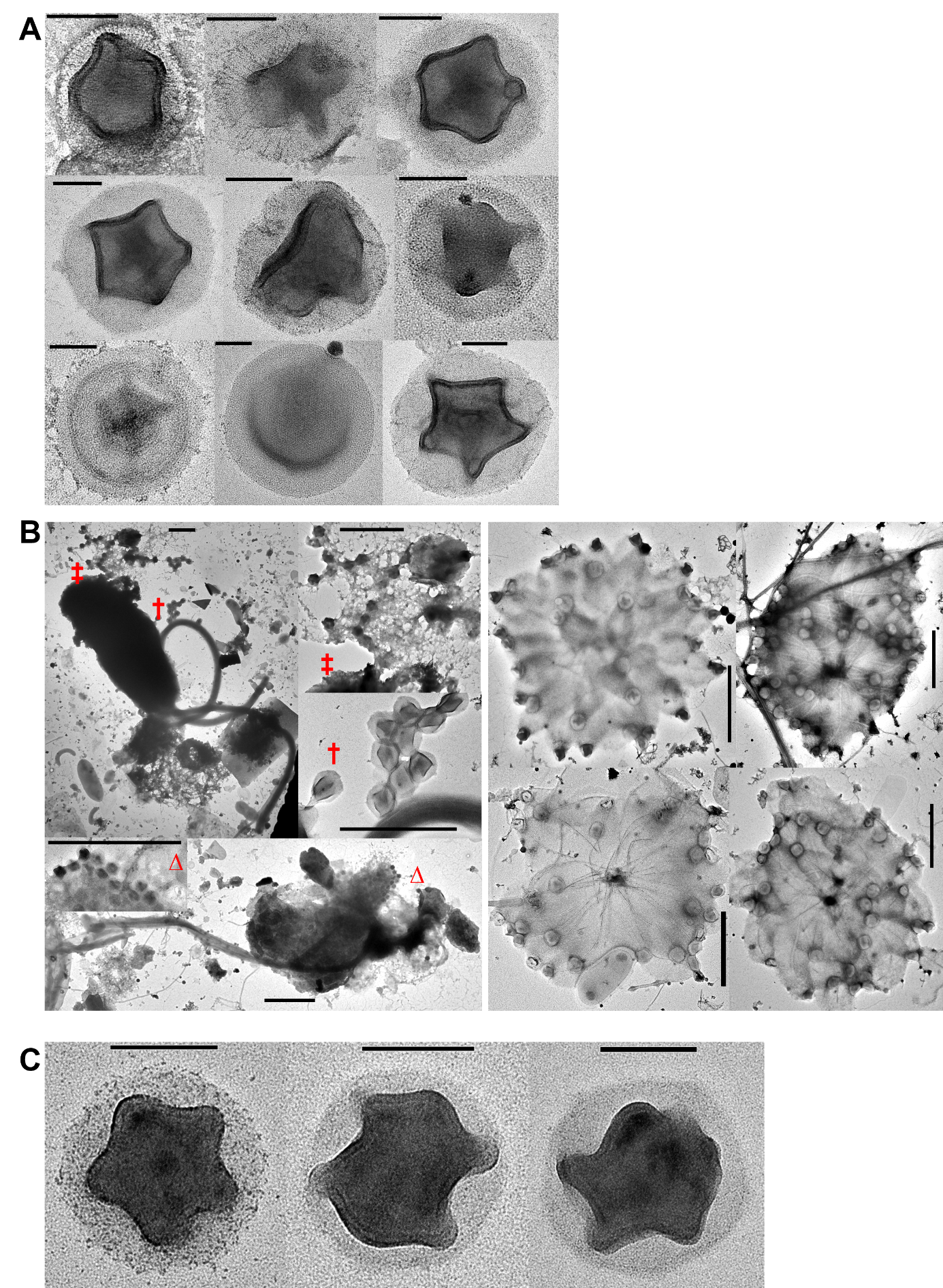


**Supplementary figure 2** **A**, Negative staining electron micrographs of various Christmas star (CS) virus-like particles (VLPs) Scale bars= 100 nm. **B**, Negative staining electron micrographs of CS VLPs derived from lytic events of flagellated microeukaryote or amoeboid form hosts. Scale bars = 1µm. **^‡†^** Illustrated zoom parts. **C**, Negative staining electron micrographs of CS VLPs detected in marine environment. Scale bars = 100 nm.


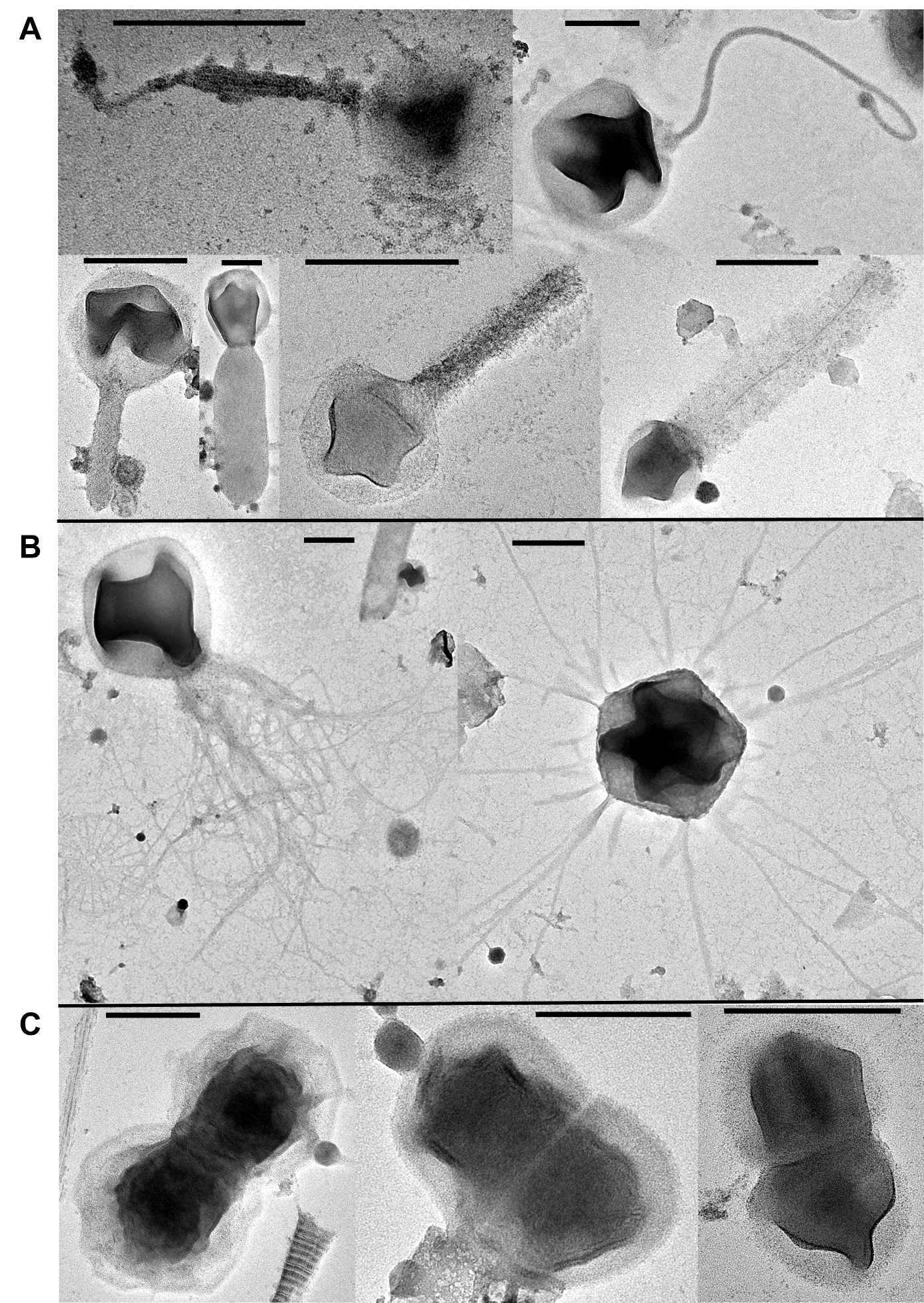


**Supplementary figure 3** Negative staining electron micrographs of Christmas star (CS) virus-like particles (VLPs) detected in eutrophic French lakes. **A**, Tailed CS VLPs. **B**, Fibrillar CS VLPs. **C**, Tandem CS VLPs. Scale bars = 200 nm.


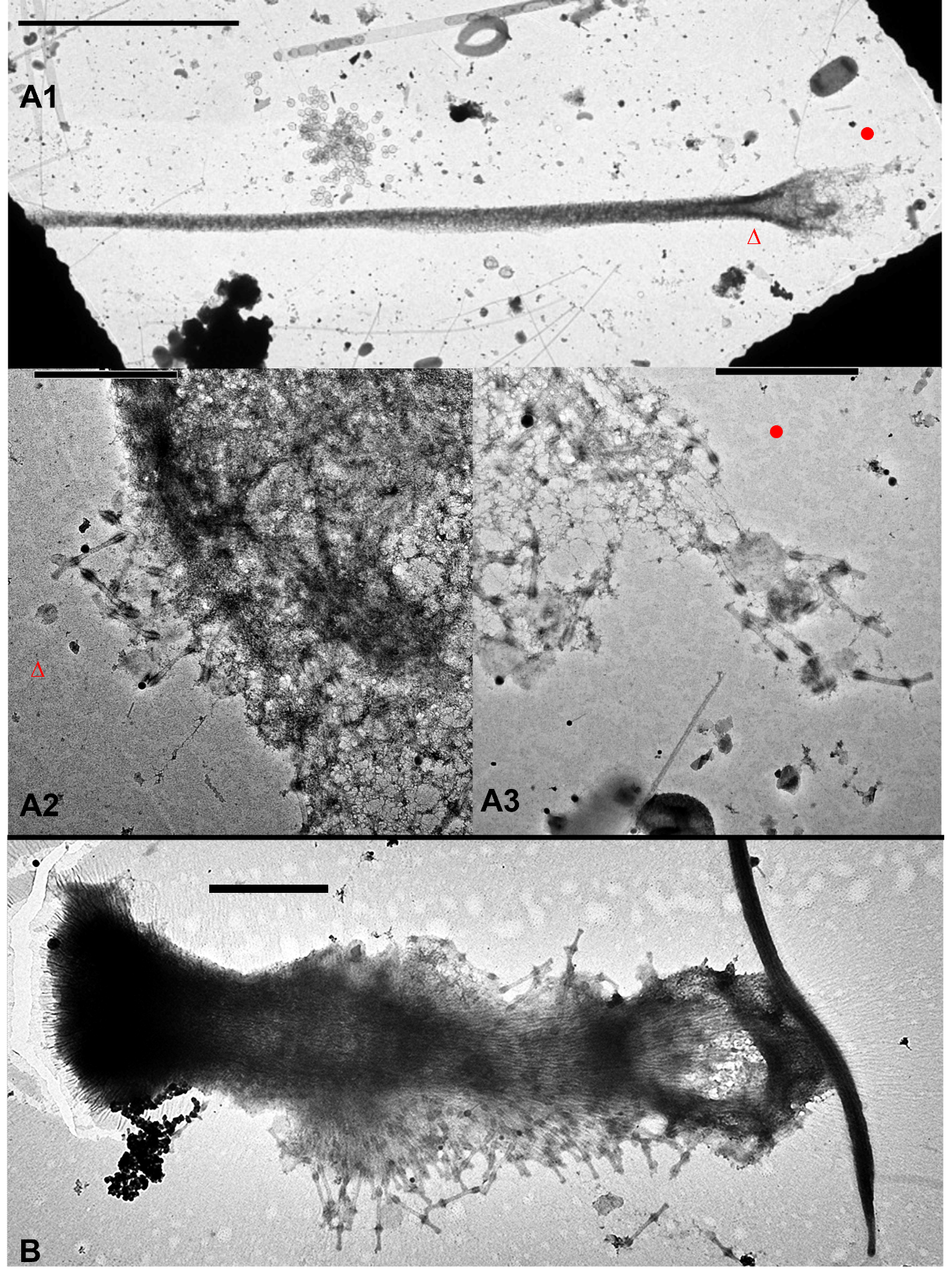


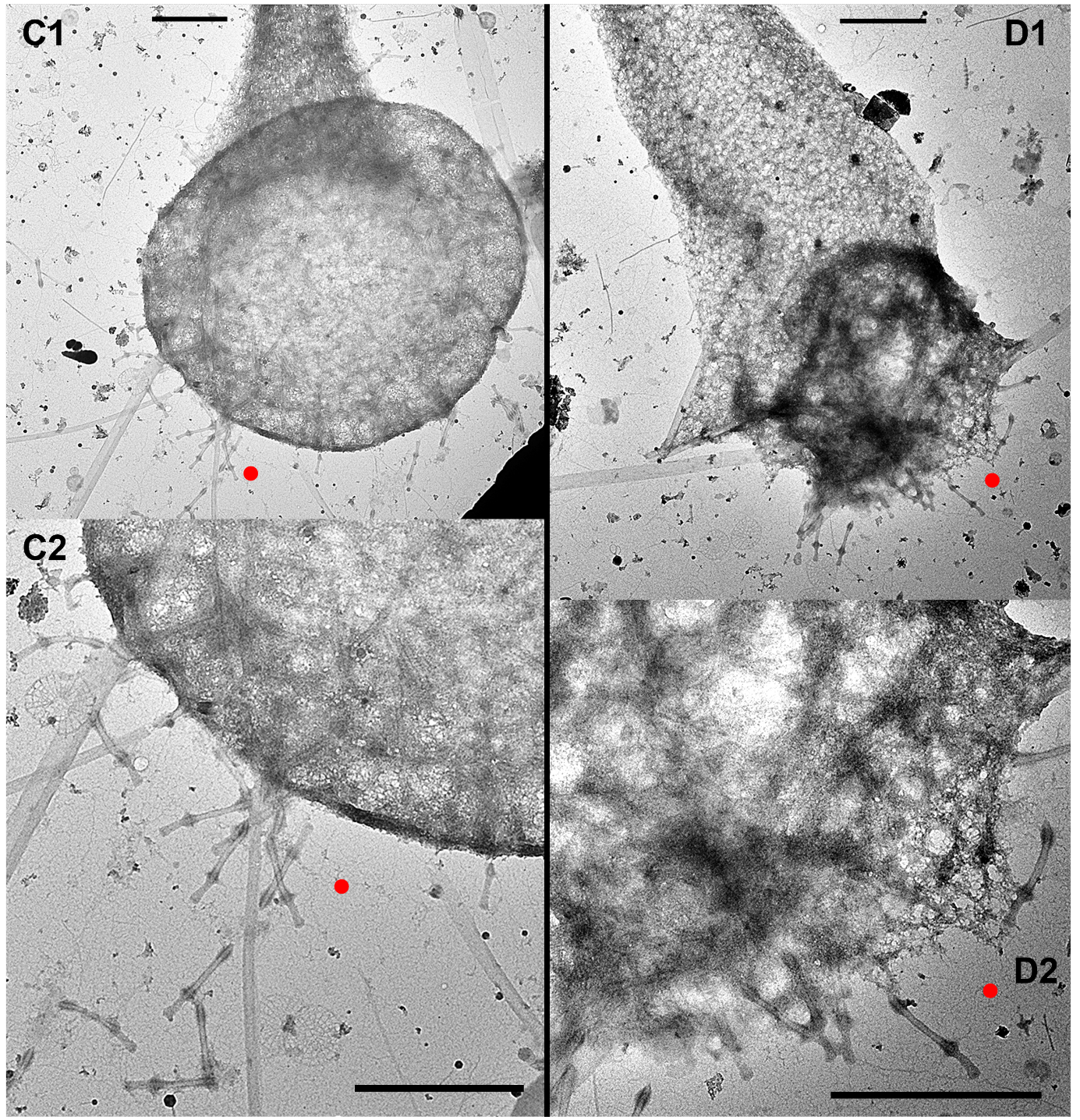


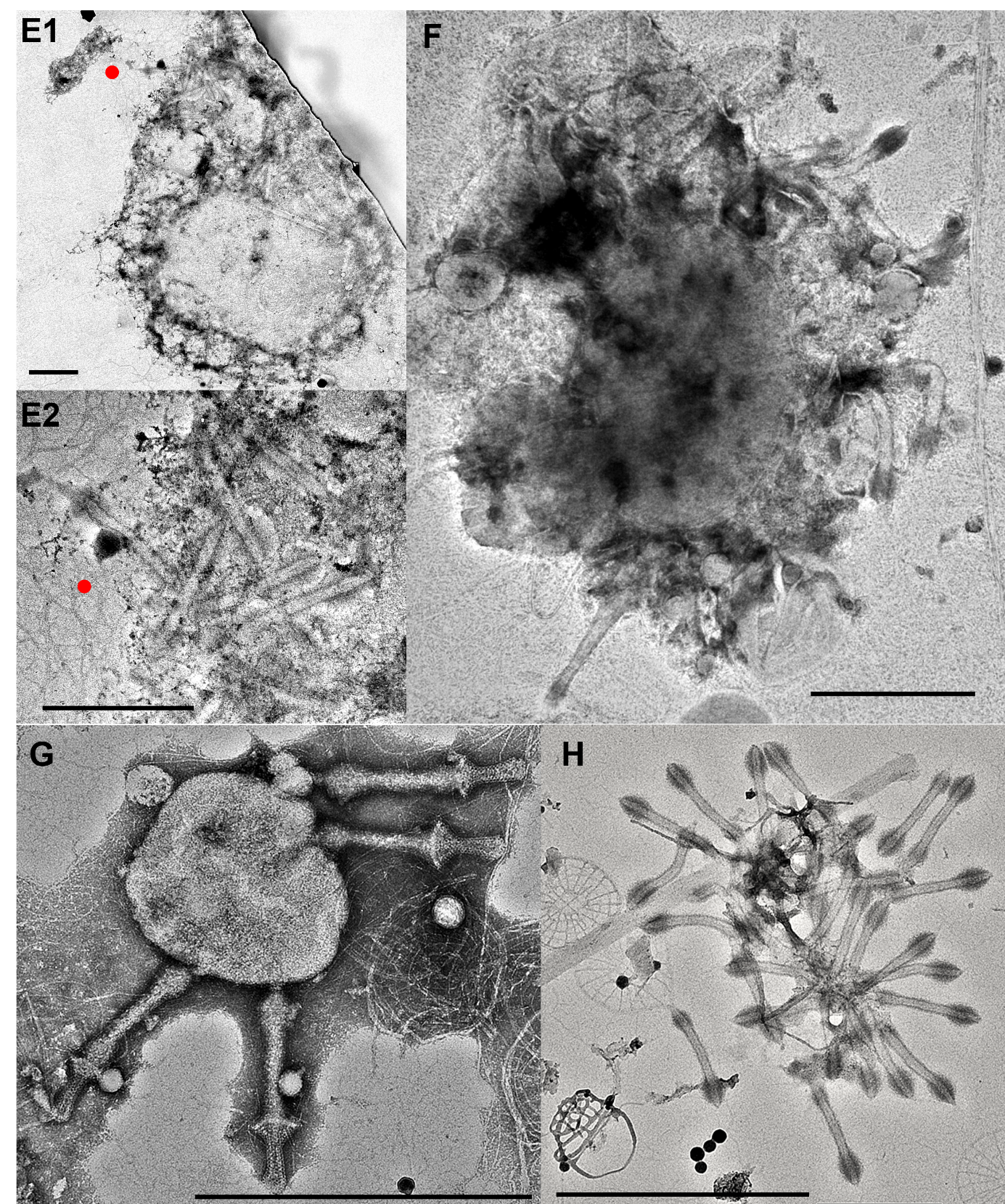
**Supplementary figure 4** Putative interactions between Sword virus-like particles (VLPs) and heterotrophic microeukaryotes (HMs). **A1** (zoom part **A2** and **A3)**, **B**, **C1** (zoom part **C2**), **D1** (zoom part **D2**), and **E1** (zoom part **E2**) negative staining electron micrographs of Sword VLPs detected in a burst HM. **F**, **G** and **H**, negative staining electron micrographs of Sword VLPs released soon after burst events. Some Sword VLPs as in **H** are broken or not fully assembled, i.e. only showing the tail. **^∆•^** Illustrated the location of the zoom parts. Scale bars A1 = 10 µm; A2, B2, B, C1, C2, D1, D2, G, H = 1 µm; E1, E2, F = 500 nm.


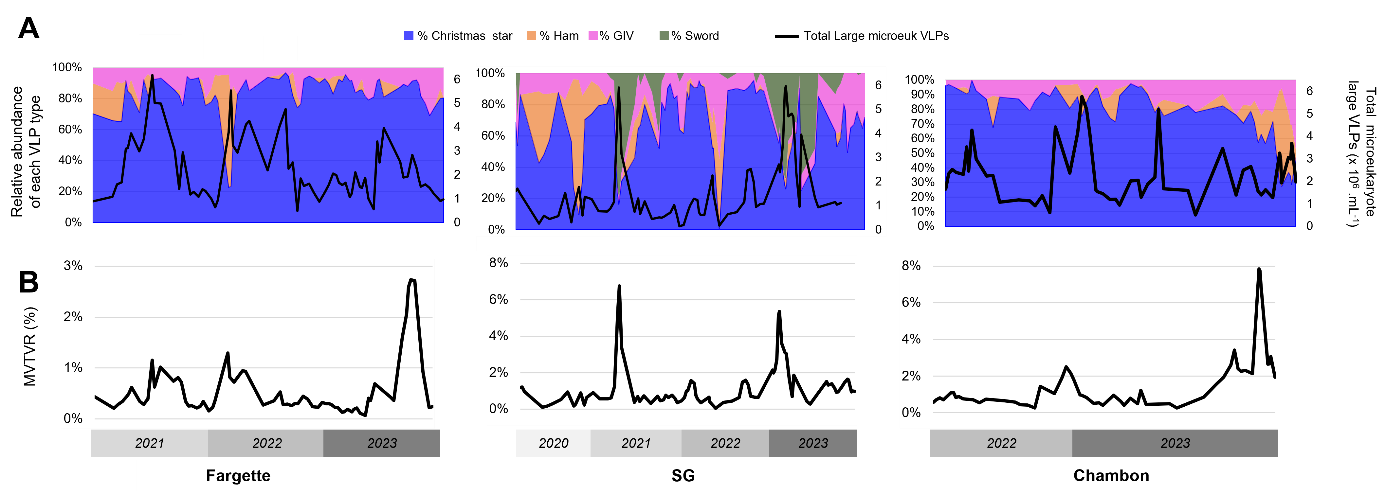
**Supplementary figure 5** Seasonal dynamics in lakes Fargette, SG and Chambon (n = 71, 81, 53 respectively) of relative abundances of Christmas star, Sword, Ham, GIV and total microeukaryote large virus-like particles (VLPs) abundance **(A)** and of the large microeukaryote VLPs to total virus ratio (MVTVR) **(B)**. Each data was obtained from the average of triplicates. Only data for which counts of all viruses-like particles considered are available are displayed.


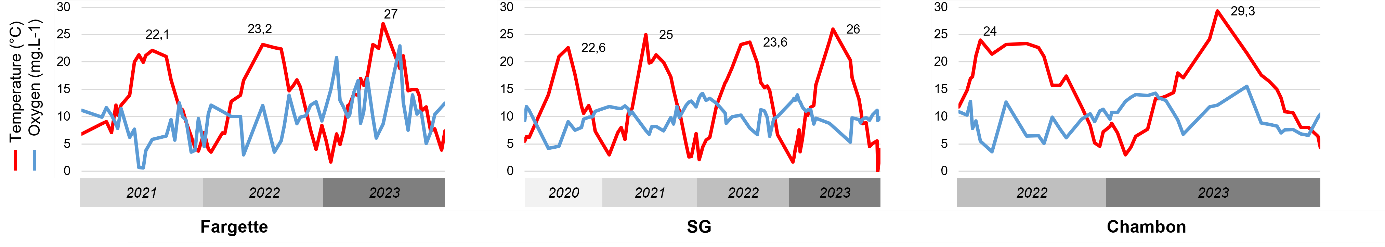


**Supplementary figure 6** Seasonal dynamics of temperature and oxygen contents in lakes Fargette, SG and Chambon (n = 71, 81, 53 respectively). Only data for which counts of all viruses-like particles considered are available are displayed.


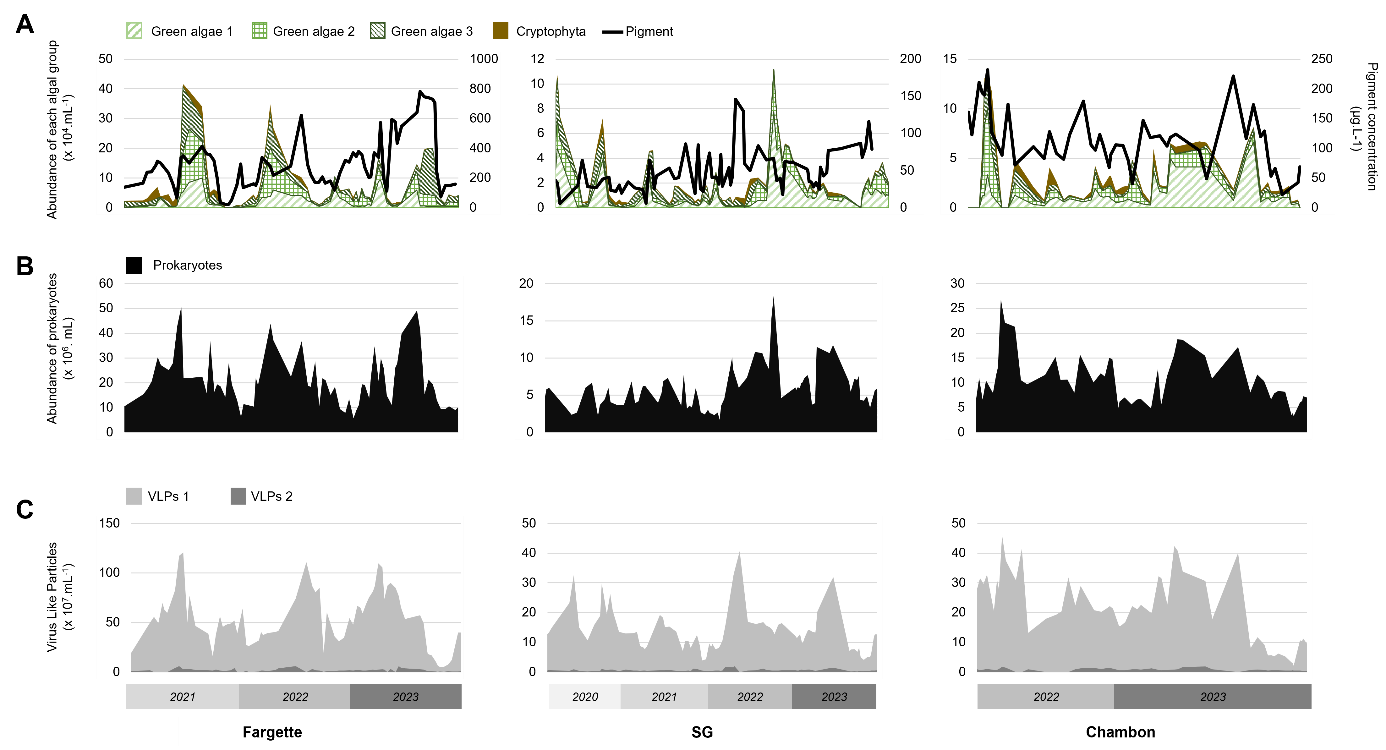


**Supplementary figure 7** Seasonal dynamics of autotrophic microeukaryotes abundance and total pigment concentration (**A**), prokaryotes abundance (**B**), and VLPs 1 and 2 respectively abundances (**C**) in lakes Fargette, SG and Chambon (n = 71, 81, 53 respectively). Each data, excepted autotrophic microeukaryotes, represent the average of triplicates. Only data for which counts of all viruses-like particles considered are available are displayed.


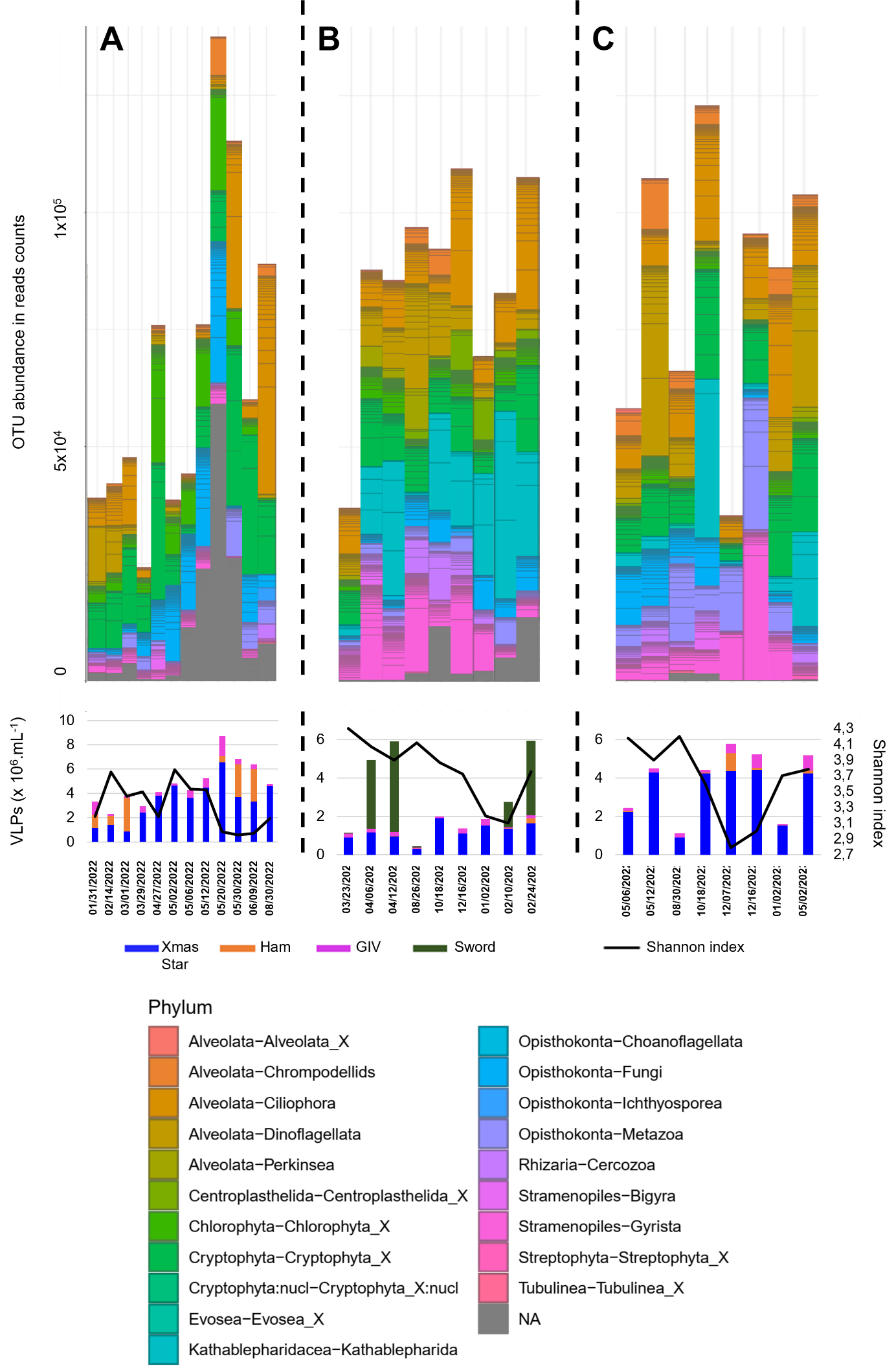


**Supplementary figure 8** Temporal dynamics of eukaryote OTU abundance in reads counts, and of eukaryote diversity index (Shannon index) compare to viral abundance of Ham, Christmas star, Sword and giant icosahedral viruses (GIV) in lakes Fargette **(A)**, SG **(B)** and Chambon **(C)**.
