# Supplementary material for: Unexpected diversity and ecological significance of uncultivable large virus-like particles in aquatic environments
Source: ISME Commun. 2025 Jun 5;5(1):ycaf098. doi: 10.1093/ismeco/ycaf098 (PMC12204322; doi:10.1093/ismeco/ycaf098)
Supplement: Supplementary_Notes_Billard_et_al_2025_ycaf098 [file supplementary_notes_billard_et_al_2025_ycaf098.docx]

**Supplementary notes**

**Unexpected diversity and ecological significance of uncultivable large virus-like particles in aquatic environments**

Hermine Billard, Maxime Fuster, François Enault, Jean-François Carrias, Léa Fargette, Margot Carrouée, Perrine Desmares, Tom O. Delmont, Pauline Nogaret, Estelle Bigeard, Gwenn Tanguy, Anne-Claire Baudoux, Urania Christaki, Télesphore Sime-Ngando, Jonathan Colombet*

Affiliations

Laboratoire Microorganismes : Génome et Environnement (LMGE), UMR CNRS 6023, Université Clermont-Auvergne, F-63000 Clermont-Ferrand, France

Hermine Billard, Maxime Fuster, François Enault, Jean-François Carrias, Léa Fargette, Margot Carrouée, Perrine Desmares, Télesphore Sime-Ngando, Jonathan Colombet

Génomique Métabolique, Genoscope, Institut François Jacob, CEA, CNRS, Univ. Evry, Université Paris-Saclay, Evry, France

Tom O. Delmont

Sorbonne Université, CNRS, Station Biologique de Roscoff, FR2424, Roscoff, France

Gwenn Tanguy

Sorbonne Université, CNRS, Station Biologique de Roscoff, UMR 7144, Roscoff, France

Estelle Bigeard, Pauline Nogaret, Anne-Claire Baudoux

UMR CNRS 8187 LOG, Université Littoral Côte d’Opale, Université de Lille, Wimereux, France

Urania Christaki

*Corresponding author: Jonathan Colombet.

Email : [jonathan.colombet@uca.fr](mailto:jonathan.colombet@uca.fr)

**Contents**

1. **Study sites and sample collection**
2. **Abiotic parameter measurements**
3. **Biotic parameter analysis**
4. **Study sites and sample collection**

Samples were collected at the surface (0-40 cm) of three artificial freshwater lakes: Fargette (45°44’39’’N; 3°27’21’’E; 465 m altitude; surface area 1.2 ha; maximum depth 2.5 m), Saint Gervais d’Auvergne (SG) (46°02’15’’N; 2°48’43’’E; 680 m altitude; surface area 10.5 ha; maximum depth 4.5 m), and Chambon (45°50’22’’N; 3°30’17’’E; 490 m altitude; surface area 1.2 ha; maximum depth 6 m). These lakes are located within a 120 km radius in the French Massif Central. Fargette is a hyper-eutrophic lake, while SG and Chambon are eutrophic lakes with a significant human presence (leisure, fishing, swimming, etc.). At regular intervals, we sampled Lake Fargette from December 21, 2020, to January 18, 2024, Lake SG from February 13, 2020, to January 18, 2024, and Lake Chambon from March 23, 2022, to January 18, 2024, sampling a total of 86, 104, and 62 time points, respectively.

Fixed samples with 1% (v/v) formaldehyde intended for the counts and determination of virus-like particles (VLPs), prokaryotes (FC, TEM), and autotrophic/heterotrophic eukaryote communities (by light microscopy) were stored at 4^◦^C until analysis. Unfixed samples intended for the analysis of autotrophic eukaryote communities by FC and for diversity analysis were stored at 4°C until processing in the 4 hours following sampling.

Marine samples were also collected from North Atlantic waters during the APERO expedition onboard *Pourquoi Pas?* in June and July 2023. One-liter volumes were collected from Nisking bottles at a depth of 2, 20, and 200 m at the three stations (PSS1: 48°27,167 N; 22°30,059 W, PSS2: 50°37,250 N; 19°7,098 W, PSS3: 47°49,846 N; 15°46,690 W). The water was prefiltered through 1.2 µm glass-fibers filters (GFC Whatman) and concentrated 9- to 10-fold by ultrafiltration using a 0.2 µm cartridge (PES Vivaflow, Sartorius). An 8 mL aliquot of the concentrate was fixed with EM grade glutaraldehyde (2% final concentration), flash frozen, and stored at –80°C until analysis.

1. **Abiotic parameter measurements**

Dissolved oxygen content (mg.L^-1^) and temperature (°Celsius) were measured *in situ* with a submersible probe (ProDSS YSI, Yellow Springs, Ohio, USA).

1. **Biotic parameter analysis**

*Total pigment analysis (probe) and phytoplankton count (flow cytometry)*

Total pigment content was measured (μg.L^-1^) using a submersible spectrofluorometric probe (BBE FluoroProbe, Moldaenke GmbH, DE) directly placed in the lake. Counts of pico- and nanophytoplankton populations (green algae, Cyanobacteria, Cryptophyta) were determined by FC using a BD LSR Fortessa X-20 (BD Sciences, San Jose, CA). Autotrophic organisms were categorized into five subpopulations (three subpopulations of green algae, Cyanobacteria, and Cryptophyta) according to their pigment content. Fluorescence signals from chlorophyll, phycoerythrin, and phycocyanin were collected using 405nm (50mW), 561nm (50mW), and 640nm (40 mW) lasers and 670/30, 586/15, and 670/14 filters, respectively. Green algae correspond to cells containing only chlorophyll. Cyanobacteria is the sum of phycoerythrin- and phycocyanin-rich cyanobacteria (here, we combined picocyanobacteria and large cyanobacteria). Finally, Cryptophyta corresponds to populations whose main pigments are phycoerythrin and chlorophyll.

*Community composition of a**uto- and heterotrophic microeukaryotes (light microscopy)*

The community composition of microeukaryotes, including eukaryotic algae, heterotrophic flagellates, and ciliates, was assessed by light microscopy on selected samples. Subsamples of 1 to 4 ml (taken from the 1% formaldehyde-fixed samples) were inoculated in Utermöhl’s settling chambers containing 10 ml of < 2µm-distilled water to ensure a proper dispersion of the settled cells on the slide. The next day, slides were examined and counted under inverted epifluorescence microscopy (Zeiss Axiovert 200M, Carl Zeiss company, Oberkochen, Germany) from randomly selected transects. Cells were visualized at x400 magnification and pigmented taxa were distinguished by detecting the autofluorescence of chlorophyll a and phycoerythrin under blue light (450–490 nm) and green light (520–560 nm) excitation, respectively. All the taxa were identified with respect to their size, shape, and specific morphological characteristics (e.g., presence of flagella or cilia, colonial forms, autofluorescence, shell) to the lowest possible taxonomic level.

*Diversity of eukaryotes (18S metabarcoding analysis)*

For a selection of unfixed samples, microbial communities were collected on a 0.2-µm polycarbonate filter (Millipore) (until saturation, pressure < 25 kPa) and stored at –20°C until DNA extraction. The filters were covered with a lysing buffer (lysozyme 2 mg ml-1, SDS 0.5%, Proteinase K 100 µg mL-1, and RNase A 8.33 µg mL-1 in TE buffer pH 8) at 37°C for 90 min. A CTAB 10% / NaCl 5 M solution was added, and the samples were incubated at 65°C for 30 min. Nucleic acids were extracted with phenol–chloroform–isoamyl alcohol (25:24:1); the aqueous phase containing the nucleic acids was recovered and purified by adding chloroform-isoamyl alcohol (24:1). Nucleic acids were then precipitated with a mixture of glycogen 15 µg mL-1, sodium acetate 0.1M, and ethanol 100% overnight at –20 °C. The DNA pellet was rinsed with ethanol (70%), dried, and dissolved in the TE buffer. DNA was then purified using a commercial kit (NucleoSpin® gDNA Cleanup, Macherey-Nagel) and quantified by Qubit dsDNA HS kit.

The V4 region of the eukaryote SSU RNA gene was amplified using the TAReuk454FWD1 (5’-CCAGCASCYGCGGTAATTCC-3’) and TAReukREV3 (5’- ACTTTCGTTCTTGATYRA-3’) primers tagged with adaptors as recommended by the sequencing platform. Each polymerase chain reaction (PCR) was performed in a total volume of 50 µL containing 1 × final reaction buffer, 2 mM MgCl2, 0.2 mM dNTP, 250 µg mL-1 BSA, 0.4 µM each primer, 1.25 U GoTaq ® Flexi DNA Polymerase (PROMEGA), and 20 ng DNA template. The PCR protocol used an initial activation step at 94°C for 1 min, followed by 12 “three-step” cycles consisting of 94°C for 10 s, 53°C for 30 s, and 72°C for 30 s, followed by a further 18 “three-step” cycles consisting of 94°C for 10 s, 48°C for 30 s, and 72°C for 30 s, and a final 10-min extension at 72°C. PCR products were purified on agarose gel with Nucleospin® Gel and the PCR clean-up kit (Macherey-Nagel) and quantified using the Qubit dsDNA HS kit.

Library preparation, sequencing (on Illumina MiSeq, v3, 2*300 cycles), and metabarcoding bioinformatic analysis (reference database: https://pr2-database.org/) were performed by the sequencing platform (Microsynth, Switzerland).
