# Supplementary material for: Unexpected diversity and ecological significance of uncultivable large virus-like particles in aquatic environments
Source: ISME Commun. 2025 Jun 5;5(1):ycaf098. doi: 10.1093/ismeco/ycaf098 (PMC12204322; doi:10.1093/ismeco/ycaf098)
Supplement: Supplementary_results_Billard_et_al_2025_ycaf098 [file supplementary_results_billard_et_al_2025_ycaf098.docx]

**Supplementary results**

**Unexpected diversity and ecological significance of uncultivable large virus-like particles in aquatic environments**

Hermine Billard, Maxime Fuster, François Enault, Jean-François Carrias, Léa Fargette, Margot Carrouée, Perrine Desmares, Tom O. Delmont, Pauline Nogaret, Estelle Bigeard, Gwenn Tanguy, Anne-Claire Baudoux, Urania Christaki, Télesphore Sime-Ngando, Jonathan Colombet*

Affiliations

Laboratoire Microorganismes : Génome et Environnement (LMGE), UMR CNRS 6023, Université Clermont-Auvergne, F-63000 Clermont-Ferrand, France

Hermine Billard, Maxime Fuster, François Enault, Jean-François Carrias, Léa Fargette, Margot Carrouée, Perrine Desmares, Télesphore Sime-Ngando, Jonathan Colombet

Génomique Métabolique, Genoscope, Institut François Jacob, CEA, CNRS, Univ. Evry, Université Paris-Saclay, Evry, France

Tom O. Delmont

Sorbonne Université, CNRS, Station Biologique de Roscoff, FR2424, Roscoff, France

Gwenn Tanguy

Sorbonne Université, CNRS, Station Biologique de Roscoff, UMR 7144, Roscoff, France

Estelle Bigeard, Pauline Nogaret, Anne-Claire Baudoux

UMR CNRS 8187 LOG, Université Littoral Côte d’Opale, Université de Lille, Wimereux, France

Urania Christaki

*Corresponding author: Jonathan Colombet.

Email : [jonathan.colombet@uca.fr](mailto:jonathan.colombet@uca.fr)

**Contents**

1. **Overlooked diversity and ecological significance of jumbo phages**
2. **Supplementary results figure 1**
3. **Supplementary results figure 2**
4. **References**
5. **Overlooked diversity and ecological significance of jumbo phages**

Although a number of jumbo phages were previously isolated and cultivated[1, 2], their diversity and ecology in aquatic environments were almost exclusively analyzed by metagenomics[3, 4]. These studies uncovered an enormous diversity, suggesting that jumbo phages are important, yet underestimated components of microbial communities and food webs[3]. To examine this viral component, we identified a specific cytometric population that corresponds to jumbo phage-like particles, as confirmed by TEM observations following FC sorting (**Supplementary results fig. 1A**). The population, named jumbo, was characterized by a low SSC and a high level of fluorescence, which is a proxy for the large genome sizes of jumbo phages (> 200 kb) and consistent with what was previously demonstrated for the iconic jumbo coliphage T4[5]. TEM showed capsid sizes between 90 and 110 nm in diameter, with total lengths between 270 and 467 nm (**Supplementary results fig. 1A-B**) illustrating the diversity of the jumbo phages. The overall organization of jumbo phages was characterized by head-tailed phages of the class *Caudoviricetes*[1]. Jumbo phages reached 7.9 x 10^6^ VLPs.mL^-1^, and their dynamics showed irregular phases of development (**Supplementary results fig. 1C**). For example, there was a noticeable peak in Lake SG from March 8 to July 4, 2022. Jumbo phage-like particles were detected in all the years under investigation. As expected, this population showed a positive correlation with prokaryotes (R² = 0.63, p-value < 0.00001).

The potential jumbo phages observed in the raw environmental samples (**Supplementary results fig. 1B and supplementary results fig. 2**) were again more morphologically diverse than those belonging to the sorted jumbo population. Whereas some particles consisted of a well-defined capsid and tail, others had tails that were covered by complex sheath-like structures or that were remarkably long, up to 2,200 nm in length. Even though we cannot exclude the possibility that all these diverse tailed particles were in our defined jumbo cytometric gate, most were likely rare and outside any cytometric gate. The affiliation of these rare particles as phages (**Supplementary results fig. 2B and C**) will need to be verified in future work.

These results show that jumbo phages are neglected in the aquatic environment in terms of their diversity, abundance, and induced mortality. They could be of major importance in the microbial loop[3].

1. **Supplementary results figure 1**


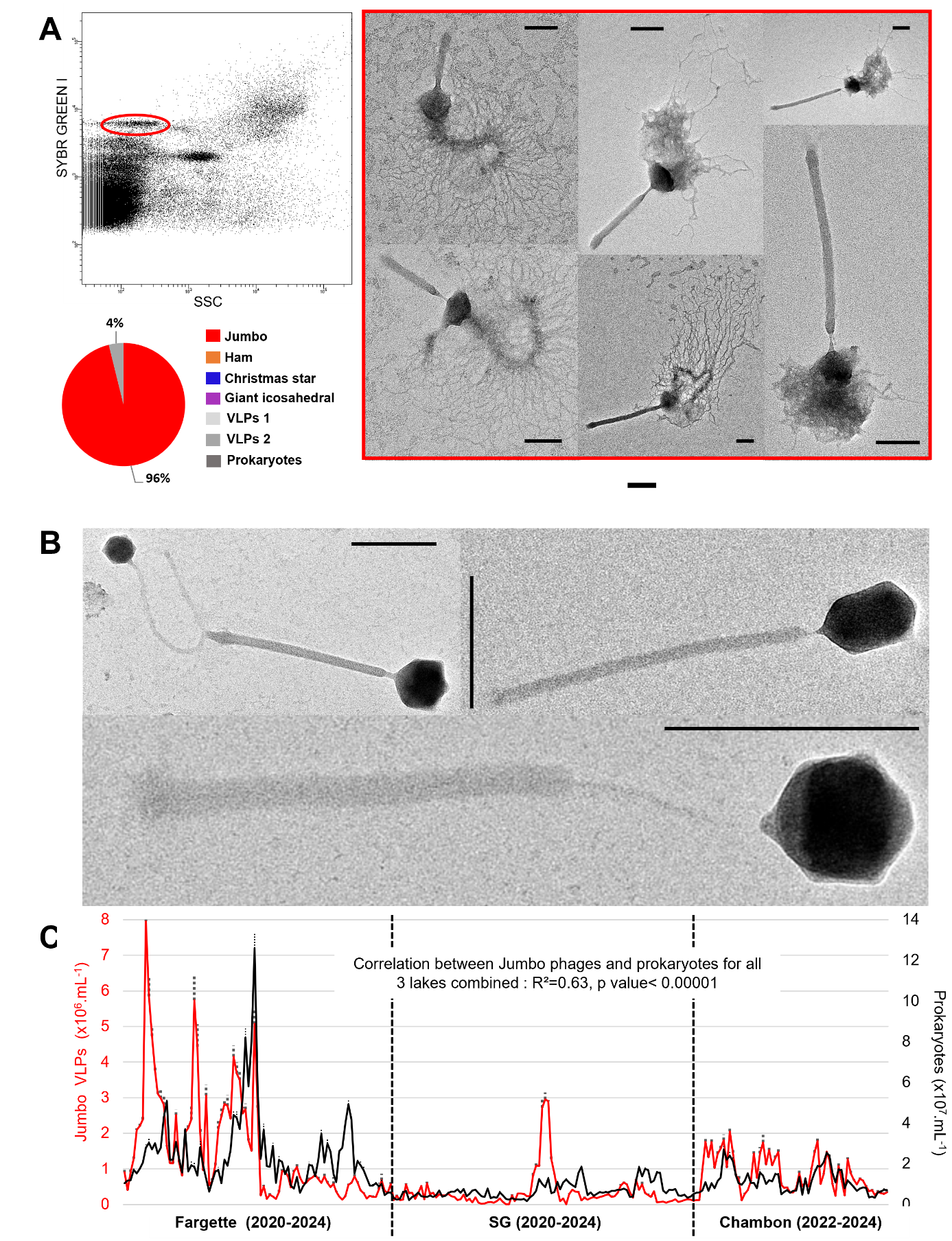


**Supplementary results figure 1.** Detection, morphological and ecological characterization of jumbo-like phage populations identified in eutrophic French lakes. **A,** Flow cytometry (FC) detection of a jumbo-like phage population (red circle) and pie chart of diversity of entities recorded by transmission electron microscopy after FC sorting in the corresponding population with micrographs of the jumbo-like phage sorted. Note the deleterious effect of sorting with visible damage on the capsid of jumbo-like phages on which the molecular structure comes out of the head. Note the heterogeneity in tail size of the jumbo phages in this population illustrating the diversity of these jumbo phages. **B**, Negative staining electron micrographs of jumbo-like phages detected in environmental samples without using FC sorting. Scale bars in A = 100 nm, B = 200 nm. **C**, Seasonal dynamics of jumbo-like phage and prokaryote abundances, in lakes Fargette, SG and Chambon. Each data represents the average of triplicates, dotted lines indicate standard deviation. n = 252.

1. **Supplementary results figure 2**


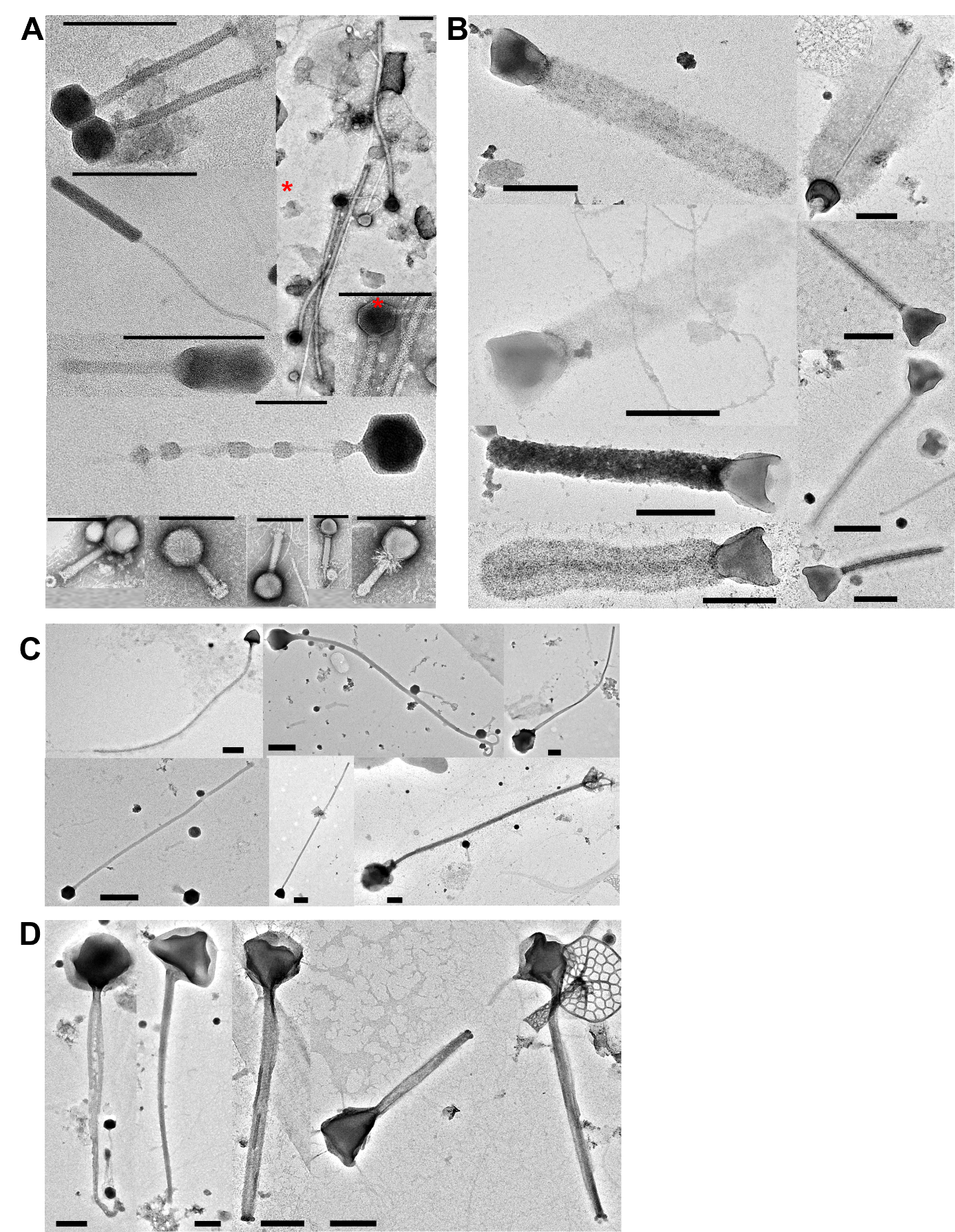


**Supplementary results figure 2.** Negative staining electron micrographs of tailed virus-like particles (VLPs) detected in eutrophic French lakes.  **A,** jumbo-like phages. **B,** sheath tailed VLPs. **C,** naked elongated VLPs. **D,** tubular tailed VLPs. Scale bars = 200 nm.

1. **References**

1. Yuan Y, Gao M. Jumbo Bacteriophages: An Overview. *Front Microbiol* 2017;**8**. https://doi.org/10.3389/fmicb.2017.00403

2. Nazir A et al. Emerging Aspects of Jumbo Bacteriophages. *IDR* 2021;**Volume 14**:5041–5055. https://doi.org/10.2147/IDR.S330560

3. Weinheimer AR, Aylward FO. Infection strategy and biogeography distinguish cosmopolitan groups of marine jumbo bacteriophages. *The ISME Journal* 2022;**16**:1657–1667. https://doi.org/10.1038/s41396-022-01214-x

4. Al-Shayeb B et al. Clades of huge phages from across Earth’s ecosystems. *Nature* 2020;**578**:425–431. https://doi.org/10.1038/s41586-020-2007-4

5. Brussaard CPD. Optimization of Procedures for Counting Viruses by Flow Cytometry. *Appl Environ Microbiol* 2004;**70**:1506–1513. https://doi.org/10.1128/AEM.70.3.1506-1513.2004
